# Mitophagy in the pathogenesis and management of disease

**DOI:** 10.1038/s41422-025-01203-7

**Published:** 2026-01-05

**Authors:** Qi Wang, Yu Sun, Terytty Yang Li, Johan Auwerx

**Affiliations:** 1 Laboratory of Integrative Systems Physiology, Interfaculty Institute of Bioengineering, École Polytechnique Fédérale de Lausanne, Lausanne, Switzerland; 2 Shanghai Key Laboratory of Metabolic Remodeling and Health, Laboratory of Longevity and Metabolic Adaptations, Institute of Metabolism and Integrative Biology, Fudan University, Shanghai, China

**Keywords:** Autophagy, Cell signalling

## Abstract

Mitophagy, an evolutionarily conserved quality-control process, selectively removes damaged mitochondria to maintain cellular homeostasis. Recent advances in our understanding of the molecular machinery underlying mitophagy — from receptors and stress-responsive triggers to lysosomal degradation — illustrate its key role in maintaining mitochondrial integrity and adapting mitochondrial function to ever-changing physiological demands. In this review, we outline the fundamental mechanisms of mitophagy and discuss how dysregulation of this pathway disrupts mitochondrial function and metabolic balance, driving a wide range of disorders, including neurodegenerative, cardiovascular, metabolic, and immune-related diseases, as well as cancer. We explore the dual role of mitophagy as both a disease driver and a therapeutic target, highlighting the efforts and challenges of translating mechanistic insights into precision therapies. Targeting mitophagy to restore mitochondrial homeostasis may be at the center of a large range of translational opportunities for improving human health.

## Introduction

Mitochondria are central metabolic hubs essential for cellular homeostasis. In addition to their well-known role as powerhouses of the cell, generating ATP through oxidative phosphorylation (OXPHOS), mitochondria also regulate fatty acid metabolism,^1^ calcium homeostasis,^2^ reactive oxygen species (ROS) generation,^3^ innate immune signaling,^4,5^ and cell death,^6^ making them important signaling integration platforms.^7–9^ Maintaining mitochondrial integrity is crucial for cellular, tissue, and organismal health, as mitochondrial dysfunction is a shared feature of aging and numerous diseases.^10,11^

Unlike other organelles, mitochondria originate from α-proteobacteria^12,13^ that symbiotically evolved within eukaryotic cells. They retain vestiges of their bacterial genome, mitochondrial DNA (mtDNA),^14^ which encodes essential components of the electron transport chain (ETC). However, most mitochondrial proteins (~1400) are nuclear encoded,^15^ requiring precise coordination between nuclear DNA (nDNA) and mtDNA to ensure proper mitochondrial function. This complex regulatory interplay subjects mitochondria to multiple levels of transcriptional, translational, and post-translational control, making them particularly susceptible to mitonuclear imbalance and proteotoxic stress.^7,8^

To maintain homeostasis, mitochondria engage in dynamic quality-control processes, including biogenesis, mitochondrial dynamics (fission and fusion), proteostasis, and stress responses such as the mitochondrial unfolded protein response (UPR^mt^).^9,16–18^ When mitochondria sustain irreparable damage, degradation pathways are activated to prevent the accumulation of dysfunctional organelles. Mitophagy is a selective form of macroautophagy that serves as the primary mechanism for eliminating damaged mitochondria through the lysosome, ensuring cellular adaptation to metabolic and environmental stresses. Additional mitochondrial clearance pathways, including mitochondria-derived vesicles (MDVs),^19,20^ mitochondrial-derived structures positive for the outer mitochondrial membrane (SPOT),^21^ vesicles derived from the inner mitochondrial membrane (VDIMs),^22^ and mitochondrial disposal via mitocytosis,^23^ have also been identified. In this review, we summarize the mechanisms of mitophagy (Supplementary information, Table S1), discuss its role in disease pathogenesis, and explore emerging therapeutic strategies that target mitophagy to restore mitochondrial homeostasis.

## Mitophagy pathways

Mitophagy was first observed in the 1970s,^24^ but specific mitophagy pathways have only recently been characterized. The selective removal of damaged or superfluous mitochondria relies on “eat me” signals, generated either by ubiquitin (Ub)-dependent or receptor-mediated pathways. In the Ub-dependent pathway, ubiquitin chains are conjugated to damaged mitochondria to recruit autophagy receptor proteins and subsequent autophagy machinery. In the receptor-mediated pathway, mitochondrially-localized receptor proteins directly engage with the autophagy machinery to initiate mitophagy. Regardless of the pathway, the targeted mitochondria are degraded by macroautophagy (hereafter autophagy), which involves the sequestration of cytoplasmic components by double-membrane autophagosomes that then deliver them to lysosomes for degradation.

### Ubiquitin-mediated mitophagy pathway

**Fig. 1:**
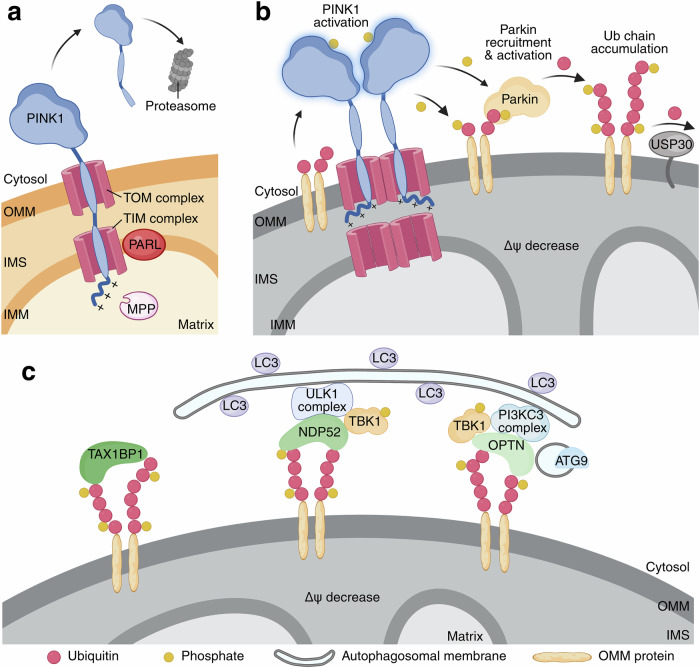
PINK1–Parkin-mediated mitophagy. **a** The rapid turnover of PINK1 under normal conditions. The N-terminus of PINK1 is imported into healthy mitochondria and processed by MPP and PARL, generating a cleaved PINK1 that is recognized by ubiquitin ligases and targeted for proteasomal degradation. **b** PINK1 accumulation on damaged mitochondria leads to Parkin recruitment and ubiquitin chain buildup. Mitochondrial damage causes mitochondrial depolarization, which inhibits PINK1 import, allowing PINK1 to complex with TOM on the OMM. Accumulated PINK1 undergoes dimerization and transactivation, phosphorylating Ser65 of the ubiquitin on OMM proteins to generate pUb. Parkin binds to these pUbs and becomes activated, decorating damaged mitochondria with ubiquitin chains. **c** Ubiquitinated mitochondria recruit selective autophagy receptors to drive mitophagy. Selective autophagy receptors such as NDP52 and OPTN bind to ubiquitin chains on the mitochondria and interact with upstream autophagy modules to initiate autophagosome formation. OMM outer mitochondrial membrane, IMS intermembrane space, IMM inner mitochondrial membrane, TOM translocase of the outer membrane, TIM translocase of the inner membrane, Δψ membrane potential.

Ubiquitination not only tags proteins for proteasomal degradation but also serves as a platform for recruiting the autophagy machinery (Box 1). The PTEN-induced kinase 1 (PINK1)–Parkin pathway, first identified through disease-causing mutations of early-onset Parkinson’s disease (PD),^25,26^ is one of the most well-characterized Ub-dependent mitophagy pathways (Fig. 1).^27^

PINK1 is a mitochondrial serine/threonine-protein kinase encoded by nDNA, translated in the cytosol, and imported into mitochondria under the guidance of its N-terminal mitochondrial targeting sequence (MTS).^28–30^ Its role as a mitochondrial damage sensor arises from its different processing in healthy versus damaged mitochondria. Under normal conditions, the 63 kDa full-length PINK1 is imported across the inner mitochondrial membrane (IMM) through the translocase of the outer membrane (TOM) and inner membrane (TIM) complexes, where the mitochondrial processing protease (MPP) cleaves off its MTS,^31^ generating a 60-kDa intermediate. This intermediate is further processed by PINK1/PGAM5-associated rhomboid-like protease (also known as Presenilin-associated rhomboid-like, PARL) to produce a ~50-kDa fragment^32–35^ that is released into the cytosol and degraded by the proteasome.^29,36,37^ Such rapid turnover ensures low endogenous PINK1 levels (Fig. 1a). By contrast, sustained mitochondrial damage or dysfunction disrupts mitochondrial membrane potential (Δψ), blocking PINK1 import and preventing its degradation. Instead, PINK1 stabilizes and accumulates on the outer mitochondrial membrane (OMM) and binds to TOM.^38–40^ If mitochondrial dysfunction is not resolved in a timely manner, persistent PINK1 accumulation leads to PINK1 dimerization and trans-autophosphorylation,^41–46^ activating PINK1 to bind and phosphorylate its substrates, serine (Ser) 65 on the ubiquitin chains of OMM proteins^47–49^ (Fig. 1b).

The accumulation of phosphorylated-ubiquitin (pUb) on the OMM recruits Parkin,^38,39,50^ a RING-between-RING E3 ligase.^51^ Recruited Parkin is subsequently phosphorylated by PINK1 at Ser65 of its ubiquitin-like domain (Ubl).^52^ While pUb binding releases Parkin from its autoinhibited state, Ubl phosphorylation further stabilizes Parkin’s open and active conformation.^38,39,52–58^ Upon activation, Parkin exhibits enhanced E3 ligase activity, ubiquitinating OMM proteins such as mitofusin 1/2 (MFN1/2), dynamin-related protein 1 (DRP1), mitochondrial Rho GTPase 1/2 (MIRO1/2, also known as RHOT1/2), and voltage-dependent anion channel 1/2 (VDAC1/2),^59–61^ thereby marking mitochondria as damaged. Additional E3 ligases also contribute to mitochondrial ubiquitination (Box 2). By contrast, USP30, a deubiquitinase (DUB) that localizes on the OMM, antagonizes Parkin-mediated mitophagy by removing ubiquitin from mitochondrial proteins.^62–64^ Notably, USP30 is also a substrate of Parkin.^62^ The newly formed ubiquitin chains are further phosphorylated by PINK1, amplifying Parkin recruitment and reinforcing a feedforward cascade (Fig. 1b). Together, PINK1 and Parkin synergize in building pUb chains that recruit Ub-binding autophagy receptors, driving autophagosome formation.

Several Ub-binding selective autophagy receptors have been characterized, including optineurin (OPTN),^65^ nuclear dot protein 52 (NDP52, also known as CALCOCO2),^66^ Tax1-binding protein 1 (TAX1BP1),^67^ p62 (also known as sequestosome 1, SQSTM1),^68^ and next to the BRCA1 gene 1 (NBR1).^69^ These receptors all contain both Ub-binding domains (UBDs) and an ATG8-interacting motif (also known as the LC3-interacting region, LIR),^68^ enabling them to recruit autophagic membranes to labeled cargo. However, arguing against the proposal that selective autophagy receptors recruit autophagy machinery through their binding to LC3, cells that lack ATG8 family LC3/GABARAP proteins can sequester mitochondria in fully formed autophagosomes,^70,71^ despite exhibiting smaller autophagosomes and a slower initial rate of autophagosome biogenesis. In line with this observation, among the five selective autophagy receptors, OPTN, NDP52, and to a lesser extent, TAX1BP1, are indispensable for mitophagy,^72–75^ whereas, p62 and NBR1 are recruited to depolarized mitochondria and are involved in mitochondrial clustering.^76,77^ More recent work suggests that OPTN and NDP52 recruit upstream autophagy modules to initiate autophagosome formation at pUb-decorated mitochondria.^78–81^ Specifically, NDP52 interacts with the ULK1 complex subunit FIP200,^78,79^ whereas OPTN recruits ATG9 vesicles that are important for de novo autophagosome biogenesis^80,81^ (Fig. 1c). In addition, TANK-binding kinase 1 (TBK1), a serine/threonine protein kinase with known functions in the innate immune pathway,^82,83^ is co-recruited to depolarized mitochondria with OPTN and becomes activated.^74,75^ Activated TBK1, in turn, phosphorylates NDP52 and OPTN at multiple sites, enhancing their binding to Ub chains^74,84^ and the autophagy complex^78^ to promote mitophagy. TBK1 also directly binds to PI3KC3 complex I, further facilitating mitophagy initiation.^85^ ATG8 family members play important roles in amplifying mitophagy^86^ and mediating autophagosome–lysosome fusion.^70,71^

Although the PINK1–Parkin pathway is well characterized in cultured cells, its relevance to mitophagy in vivo is debated. Emerging evidence suggests that basal mitophagy can proceed independently of PINK1 in vivo. *Mito*-QC is a fluorescence-based reporter system composed of a tandem mCherry–GFP fusion protein targeted to the OMM that enables the visualization of mitochondrial structure and mitophagy at single-cell resolution within tissues.^87^ A seminal study crossed *Pink1* knockout mice with *mito*-QC mice and demonstrated that *Pink1* knockout mice exhibited normal levels of basal mitophagy across multiple high-metabolic-demand tissues, with the exception of pancreatic islets.^88^ Similarly, in transgenic *Drosophila* expressing *mito*-QC or mt-Keima, another mitochondrially targeted pH-sensitive fluorescent reporter,^89^ *Pink1* or *Prkn* (the gene encoding Parkin) deficiency had minimal effects on basal mitophagy levels.^90,91^ Nonetheless, the PINK1–Parkin pathway is essential for mitophagy induction upon hypoxia exposure and rotenone treatment in *Drosophila*,^91^ and exhaustive exercise also activates PINK1–Parkin mitophagy in the mouse heart.^92^ These findings suggest that mammalian cells employ multiple pathways to regulate mitophagy and maintain mitochondrial integrity in a context-dependent manner.

Box 1 Crosstalk between autophagy and ubiquitin–proteasome systems Both autophagy and the ubiquitin–proteasome system are evolutionarily conserved catabolic processes responsible for the degradation, processing, and recycling of cellular components. Whereas autophagy primarily eliminates long-lived and insoluble protein aggregates, lipid droplets, pathogens, and dysfunctional or superfluous organelles,^439^ ubiquitin is known mainly for mediating selective proteasomal degradation of short-lived proteins.^440,441^ Recent advances in the understanding of selective autophagy mechanisms, exemplified by mitophagy, have revealed the intertwinement of the autophagy and ubiquitin–proteasome systems,^442,443^ highlighting their coordinated roles in maintaining cellular homeostasis.

Ubiquitin is a small protein consisting of 76 amino acids that serves as a molecular tag for proteasomal degradation. Proteins targeted for degradation are covalently ligated with ubiquitin following a three-enzyme cascade consisting of a ubiquitin-activating enzyme E1, a ubiquitin-conjugating enzyme E2, and a ubiquitin-protein ligase E3.^444^ First, the E1 enzyme activates ubiquitin by adenylating its C-terminal glycine residue in an ATP-dependent manner, enabling its binding to the active-site cysteine residue of E1 through a thioester linkage.^445^ The activated ubiquitin is then transferred to the active-site cysteine residue of an E2 enzyme.^446^ Finally, an E3 enzyme catalyzes the formation of an iso-peptide bond between the C terminus of ubiquitin and a lysine residue of the substrate protein^447,448^ (Fig. B1). Diverse E1, E2, and E3 proteins have been identified. The human genome is estimated to encode 2 E1s, at least 38 E2s, and over 600 E3s,^448^ among which the E3s play critical roles in determining substrate specificity and regulating elongation of the ubiquitin chain.^449^ DUBs, which are a type of protease, counteract this process by removing ubiquitin modifications.^409,441^
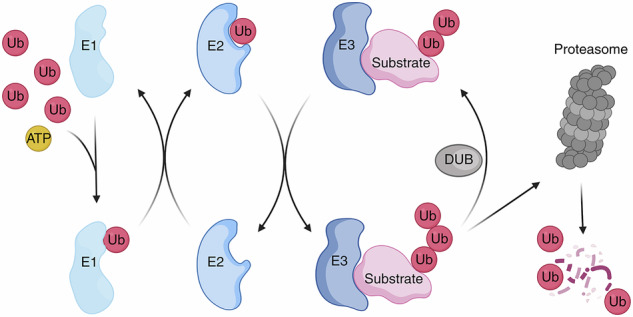

Autophagy is a lysosomal degradation pathway that includes macroautophagy, microautophagy, and chaperone-mediated autophagy. In this review, we specifically refer to macroautophagy, which can occur in either a nonselective ‘bulk’ or selective manner. The autophagy process involves several core modules: (i) The unc51-like kinase (ULK) complex, comprising ULK1 or ULK2, autophagy-related protein 13 (ATG13), ATG101, and focal adhesion kinase family kinase-interacting protein of 200 kDa (FIP200). This complex integrates upstream signals from AMP-activated protein kinase (AMPK) and the mammalian target of rapamycin complex 1 (mTORC1)^450–454^ to regulate autophagosome initiation. (ii) The transmembrane protein ATG9, which is potentially involved in lipid supply for autophagosome biogenesis.^455^ (iii) The class III phosphatidylinositol 3-kinase (PI3KC3) complex, consisting of Beclin 1, the lipid kinase VPS34, the regulatory scaffold VPS15, and ATG14.^456^ The PI3KC3 complex synthesizes phosphatidylinositol 3-phosphate (PI3P) from phosphatidylinositol (PI). (iv) The WD-repeat proteins interacting with phosphoinositides (WIPIs), which bind PI3P during early steps of autophagy and recruit the ATG16L1 complex. (v) The ATG5–ATG12/ATG16L1 complex, which acts as an E3 ligase-like ATG8 lipidation machinery.^457^ This lipidation process involves a ubiquitin-like conjugation system that also requires the E1-like enzyme ATG7^458,459^ and the E2-like ATG3.^460,461^ (vi) The ATG8 family, including microtubule-associated protein 1 A/1B-light chain 3 (LC3) and γ-aminobutyric acid receptor-associated protein (GABARAP) subfamily proteins.^462^ ATG8 proteins are conjugated to the membrane lipid phosphatidylethanolamine (PE) through a process termed lipidation, which is analogous to ubiquitination, to promote autophagosome maturation and, eventually, autophagosome–lysosome fusion.

ATG8 proteins, in addition to their classic conjugation to double membranes, can also be recruited to various single membranes, such as endosomes, phagosomes, macropinosomes, and the plasma membrane, via non-canonical pathways^463–465^ in a process termed conjugation of ATG8 to single membranes (CASM). The function of this process varies by membrane type, ranging from degradation and secretion to membrane repair. In CASM, ATG8 can be conjugated to PE, as mentioned earlier, but also to phosphatidylserine (PS).^466,467^ Notably, single-membrane lipidation shows limited reliance on classical upstream autophagy initiators (e.g., mTORC1, ULK1 complexes) but retains most canonical ubiquitin-like conjugation steps.

The identification of autophagy receptor proteins that bind both ubiquitin and components of the autophagosome assembly machinery establishes a molecular link between the autophagy and proteasome systems.^442,443,462,468^ Autophagy receptors are characterized by the presence of both a UBD and an LIR. During ubiquitin-dependent mitophagy events, UBDs recognize ubiquitinated mitochondria, while LIRs interact with autophagy machinery proteins.^468^ Such a structure enables the autophagy machinery to selectively capture ubiquitinated organelles for lysosomal degradation.

Box 2 E3 ligases in Parkin-independent mitophagy The observation that mitochondrial ubiquitin alone is sufficient to induce mitophagy, even in the absence of PINK1 and Parkin,^80^ raises the question of whether other ubiquitin ligases contribute to Parkin-independent mitophagy. Given the large number of putative human E3 ligases,^447^ it is plausible that multiple ligases participate in this process, despite Parkin being the most well-established E3 ligase in mitophagy.

Indeed, several E3 ligases have been identified as mediators of mitophagy, including mitochondrial ubiquitin ligase 1 (MUL1), seven in absentia homolog 1 (SIAH1), and ariadne RBR E3 ubiquitin ligase 1 (ARIH1). MUL1 (also known as MAPL/MULAN/GIDE) is a mitochondrial OMM-localized E3 ligase with both ubiquitination and SUMOylation activities.^469^ MUL1 has been shown to regulate mitochondrial dynamics by modifying MFN2 and DRP1.^470,471^ It modifies and interacts with ULK1,^472^ and it functions synergistically with Parkin to eliminate paternal mitochondria.^473^ SIAH1 promotes mitochondrial protein ubiquitination and subsequent mitophagy in a PINK1–synphilin-1-dependent manner^474^; ARIH1 was identified as a PINK1-dependent mitophagy regulator in cancer cells.^300^

In addition, in receptor-mediated mitophagy, HUWE1 (HECT, UBA, and WWE domain containing E3 ubiquitin protein ligase 1) regulates AMBRA1-mediated mitophagy,^155^ and MARCH5 (membrane-associated RING finger 5) ubiquitinates FUNDC1 to regulate mitophagy under hypoxic stress.^136^

### Receptor-mediated mitophagy

**Fig. 2:**
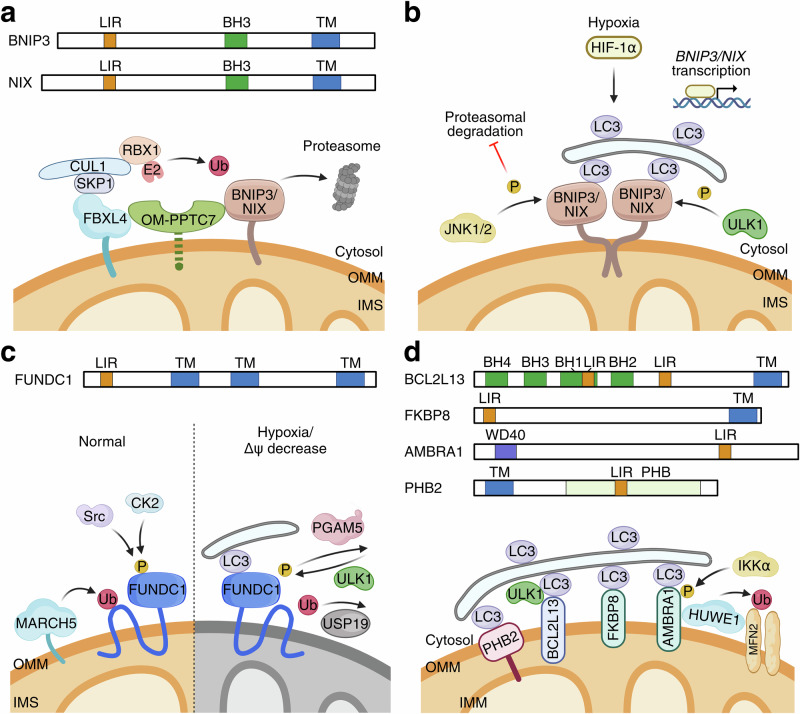
Receptor-mediated mitophagy pathways. PINK1–Parkin-independent regulatory mechanisms are shown. **a**, **b** BNIP3/NIX-mediated mitophagy. **a** Functional domains of BNIP3 and NIX are shown on top. Under normal conditions, the mitochondrial E3 ligase complex SCF^FBXL4^, which is composed of CUL1–SKP1–FBXL4, interacts with RBX1 and the E2 enzyme to ubiquitinate BNIP3 and NIX for proteasomal degradation. OM-PPTC7 facilitates the interaction between SCF^FBXL4^ and BNIP3/NIX. **b** Under hypoxia, HIF-1α upregulates the transcription of BNIP3/NIX. JNK1/2 phosphorylate BNIP3 to inhibit its degradation. Accumulated BNIP3/NIX proteins form homodimers and recruit LC3 via their LIR. ULK1 phosphorylates BNIP3/NIX to promote mitophagy. **c** FUNDC1-mediated mitophagy. Functional domains of FUNDC1 are shown on top. Under normal conditions, CK2 and Src phosphorylate FUNDC1 to prevent its interaction with LC3. The E3 ligase MARCH5 ubiquitinates FUNDC1 for proteasomal degradation, and this is antagonized by USP19. Upon hypoxia or a decrease in membrane potential, PGAM5 dephosphorylates FUNDC1 to promote its binding with LC3. ULK1 phosphorylates FUNDC1 to facilitate its LC3 recruitment. **d** BCL2L13, FKBP8, AMBRA1, and PHB2 as mitophagy receptors. Functional domains of BCL2L13, FKBP8, AMBRA1, and PHB2 are shown. These receptors mediate mitophagy by recruiting LC3 via their LIR domains. In addition, BCL2L13 complexes with ULK1 and LC3 during the initiation of mitophagy. AMBRA1 recruits the E3 ligase HUWE1 to ubiquitinate MFN2 and other OMM proteins to facilitate mitophagy, and its phosphorylation by IKKα also promotes mitophagy. PHB2, an IMM-located mitophagy receptor, acquires access to LC3 upon outer membrane rupture. OMM outer mitochondrial membrane, IMS intermembrane space, IMM inner mitochondrial membrane; LIR LC3-interacting region, BH1–4 Bcl-2 homology domain 1–4, TM transmembrane domain, WD40 WD40 domain, PHB prohibitin domain.

Mitophagy can also occur independently of ubiquitin. In this case, mitochondrial membrane proteins mediate the delivery of mitochondria to autophagosomes. These proteins, collectively termed “mitophagy receptors”, engage the autophagy machinery via their LIR domains, which interact primarily with ATG8s. Here, we focus on the key features and regulatory mechanisms of the most well-characterized receptors (Fig. 2); their physiological roles are explored in further detail elsewhere.^93–95^

#### BNIP3 and BNIP3L/NIX

BNIP3 (BCL-2/adenovirus E1B 19 kDa protein-interacting protein 3) and BNIP3L (BNIP3-like, also known as NIX) are BCL-2 homology domain 3 (BH3)-only family proteins involved in diverse cellular processes, including apoptosis and mitophagy.^96–98^ BNIP3 and NIX regulate mitochondrial clearance under various conditions.^99–104^ For example, BNIP3 mediates mitophagy under hypoxia,^99,102^ whereas NIX is essential for mitochondrial elimination during reticulocyte maturation,^100^ erythroid cell maturation,^101^ and retinal ganglion cell differentiation.^105^ Furthermore, NIX induction restores CCCP-induced mitophagy in cells derived from early-onset PD patients with PINK1 or Parkin deficiency.^106^ Both BNIP3 and NIX possess a characteristic C-terminal transmembrane domain that is responsible for homodimerization and OMM anchoring^107–109^ and an LIR domain that binds LC3/GABARAP.^104,110^ Upon activation, BNIP3 and NIX homodimerize via their transmembrane domains and are targeted to the OMM.^111^

BNIP3/NIX are regulated at both the transcriptional and post-translational levels. Hypoxia is a key stress factor that induces BNIP3/NIX-mediated mitophagy,^112^ as both genes can be transcriptionally upregulated by stabilized hypoxia-inducible factor-1alpha (HIF-1α).^113,114^ However, further investigation is needed to fully clarify the molecular mechanisms of this pathway.^115^ Post-translational modifications of BNIP3 and NIX include phosphorylation and ubiquitination. Phosphorylation of BNIP3 at Ser17 and Ser24 and of NIX at Ser34 and Ser35 promote mitophagy by stabilizing their ATG8 binding via their LIR domains.^96,116^ ULK1 phosphorylates BNIP3 at Ser17 and NIX at Ser35 to promote mitophagy.^117^ c-Jun N-terminal kinase 1/2 (JNK1/2) phosphorylate BNIP3 at Ser60 and threonine (Thr) 66 to prevent its proteasomal degradation and enhance hypoxia-induced mitophagy.^118^ The mitochondrial E3 ligase, SCF^FBXL4^, which is composed of the adaptor protein S-phase kinase-associated protein 1 (SKP1), a Cullin 1 (CUL1) backbone, and the mitochondrially localized F-box protein FBXL4, binds RING-box protein 1 (RBX1) and E2 ubiquitin-conjugating enzyme to ubiquitinate BNIP3 and NIX for proteasomal degradation.^119–121^ Protein phosphatase targeting COQ7 (PPTC7), a PP2C phosphatase localized predominantly to the mitochondrial matrix, cooperates with FBXL4 to mediate the turnover of BNIP3 and NIX, thereby regulating mitophagy.^122–125^ The PPTC7 precursor is trapped at the OMM (OM-PPTC7) by BNIP3 and NIX, where it scaffolds the assembly of the substrate–PPTC7–FBXL4 complex to degrade BNIP3 and NIX,^122,123^ forming a homeostatic regulatory loop **(**Fig. 2a, b). Starvation has been shown to upregulate PPTC7 expression in mouse liver to repress BNIP3/NIX-mediated mitophagy, which prevents starvation-induced hepatic mitochondrial and metabolic derangements.^123^

Several lines of evidence suggest that BNIP3 and NIX may also be involved in PINK1–Parkin-mediated mitophagy. BNIP3 interacts with PINK1 and inhibits its proteolytic cleavage^126^; it also regulates the mitochondrial dynamics proteins DRP1 and optic atrophy protein 1 (OPA1),^127,128^ which favor mitophagy by inhibiting fusion and promoting fission. NIX, on the other hand, is essential for uncoupler-induced mitophagy.^129^ NIX can be ubiquitinated by Parkin, and ubiquitinated NIX in turn recruits NBR1 to promote mitophagy.^130^

#### FUNDC1

FUN14 domain-containing 1 (FUNDC1) is an integral OMM protein that serves as a receptor for hypoxia-induced mitophagy.^131^ It contains three transmembrane domains and a characteristic N-terminal Tyr (tyrosine)-X-X-Leu (leucine) (Y^18^xxL^21^) LIR motif that interacts with LC3B.^131^ Post-translational modifications play a crucial role in regulating FUNDC1 function. Under basal conditions, casein kinase 2 (CK2) phosphorylates FUNDC1 at Ser13, while the tyrosine kinase Src phosphorylates FUNDC1 at Tyr18, preventing LIR–LC3B binding.^131–133^ In response to hypoxia or uncoupler-induced mitochondrial depolarization, the mitochondrial Ser/Thr protein phosphatase PGAM5 dephosphorylates FUNDC1 at Ser13, which, together with dephosphorylation at Tyr18, enhances the FUNDC1–LC3 interaction to promote mitophagy.^131–133^ In addition, ULK1 phosphorylates FUNDC1 at Ser17 under hypoxic conditions or upon mitochondrial uncoupling, further increasing LC3 binding affinity.^133,134^ In addition to its role in mitophagy, FUNDC1 also coordinates mitochondrial dynamics through its interaction with both DRP1 and OPA1. Under mitochondrial stress, such as selenite or FCCP exposure, FUNDC1 reduces its interaction with OPA1 and enhances its association with DRP1.^135^ These interactions are regulated by the phosphorylation status of FUNDC1 at Ser13.^135^ FUNDC1 stability is regulated by ubiquitination at Lys119 catalyzed by the mitochondrial E3 ligase MARCH5, whereas deubiquitination by USP19 counteracts this process, fine-tuning mitophagy^136,137^ (Fig. 2c).

#### BCL2L13

BCL2-like 13 (BCL2L13, also known as BCL-RAMBO) is an OMM-localized protein that contains several conserved BCL-2 homology domains (BH1, BH2, BH3, BH4), two LIR motifs, and a C-terminal transmembrane domain.^138^ Similar to BNIP3 and NIX, BCL2L13 was initially identified as a pro-apoptotic protein.^139^ It was later characterized as a mammalian homologue of Atg32, mediating mitochondrial fragmentation and mitophagy in a Parkin-independent manner.^138^ Upon mitophagy induction, BCL2L13 recruits LC3B to the OMM, accompanied by recruitment of the ULK1 complex, thus forming a BCL2L13–ULK1–LC3B complex^140^ (Fig. 2d). In glioblastoma, BCL2L13 is upregulated, and BCL2L13 overexpression promotes mitophagy.^141^ Mitochondrial fission may play a role in this process, as deficiency of BCL2L13 leads to reduced phosphorylation of DRP1 at Ser616, but not Ser637, corresponding to decreased DRP1 activity. In support of this notion, the DRP1-specific inhibitor Mdivi-1 suppresses BCL2L13-induced mitophagy.^141^ In addition, BCL2L13 may contribute to autophagosome formation at MERCS by acting as a mitochondrial partner.^142^ 5′-AMP-activated protein kinase catalytic subunit alpha-2 (AMPKα2) was recently shown to phosphorylate BCL2L13 at Ser272 to promote its activation.^143^ In the heart, AMPKα2 associates with BCL2L13, and pressure overload upregulates this interaction, suggesting its potential involvement in the development of cardiac dysfunction.

#### FKBP8

FKBP8 (also known as FKBP38) is a unique FKBP family member with diverse functions, including anti-apoptotic activity through anchoring BCL-2 and BCL2L1 to the mitochondrion.^144^ FKBP8 is anchored to the OMM by its transmembrane domain and recruits lipidated LC3A via its LIR domain. Carbonyl cyanide m-chlorophenylhydrazone (CCCP) enhances the binding between FKBP8 and LC3A, and co-expression of FKBP8 and ATG8 proteins, particularly LC3A, induces mitophagy^145^ (Fig. 2d). In addition, FKBP8 contains an LIR-motif-like sequence (LIRL) essential for its interaction with OPA1, suggesting potential coupling of mitochondrial fragmentation to FKBP8-mediated mitophagy.^146^ Notably, during mitophagy, FKBP8 translocates from mitochondria to the ER to escape degradation, a process that depends on the dual-localization properties of its TM domain and occurs in both Parkin-dependent and Parkin-independent manners.^145,147^ The mechanism of FKBP8 activation, its interplay with other mitophagy pathways, and its physiological significance all require further investigation.

#### AMBRA1

Activating molecule in Beclin 1 regulated autophagy protein 1 (AMBRA1) was first identified as a WD40-domain-containing protein that positively regulates autophagy and is required for Beclin 1 activity. It has a crucial role in embryonic neural development.^148^ Subsequent studies revealed its role in cell-cycle regulation through the ubiquitination and proteasomal degradation of D-type cyclins (cyclin Ds) during development and cancer.^149–151^ After mitophagy induction, AMBRA1 binds LC3 through its LIR motif, an interaction that is crucial for regulating both Parkin-dependent and -independent mitochondrial clearance.^152^ In addition, in Parkin-dependent mitophagy, the interaction between AMBRA1 and Parkin strongly increases during prolonged mitochondrial depolarization, and AMBRA1 at the perinuclear clusters of depolarized mitochondria promotes autophagosome formation by stimulating the activity of the upstream PI3K complex.^153^ On the OMM, AMBRA1 interacts with PINK1, increasing its stability by preventing its degradation by the mitochondrial protease LONP1, thereby enhancing PINK1 accumulation.^154^ Although AMBRA1 potentiates Parkin-mediated mitophagy, it can also induce mitophagy independently of Parkin, as forcing AMBRA1 localization to the mitochondria unleashes massive mitophagy in Parkin-deficient cells. Most importantly, overexpression of wild-type AMBRA1, in combination with FCCP treatment, induces mitochondrial clearance in cells that lack Parkin or PINK1.^152^ The E3 ligase HUWE1 plays a key role in this process by ubiquitinating and degrading MFN2, while also stimulating the IKKα-mediated phosphorylation of AMBRA1 at Ser1014, which increases LC3 binding^155^ (Fig. 2d). The pleiotropic roles of AMBRA1 warrant further exploration of its molecular mechanisms, pathological implications, and therapeutic potential.^156^

#### PHB2

Prohibitin 2 (PHB2) is a ubiquitously expressed protein that forms a heterodimeric complex with PHB1. These dimers assemble into a ring-like structure on the IMM.^157,158^ The PHB complex regulates membrane-protein degradation, cristae morphogenesis, mitochondrial genome stability, and the functional integrity of mitochondria.^159,160^ A recent study suggested that PHB2 is an IMM-resident mitophagy receptor during Parkin-mediated mitophagy.^161^ Upon CCCP-induced mitochondrial depolarization or inhibition of mitochondrial respiration induced by oligomycin and antimycin (OA), and in the presence of Parkin, OMM rupture exposes the PHB1/2 heterodimer, enabling its interaction with LC3-II through an LIR domain of PHB2.^161^ Knockdown of PHB2 disrupts the association between the phagophore and the IMM. Consistent with this scenario, knockdown of PHB2 or mutation of the PHB2 LIR domain abolishes OA-induced mitophagy. These findings suggest that PHB2 is essential for autophagosomal sequestration of damaged mitochondria in Parkin-mediated mitophagy^161^ (Fig. 2d). Notably, although proteasome-dependent OMM rupture is required for the PHB2–LC3 interaction, PHB2 ubiquitination was not detected, and purified PHB2 binds LC3 in vitro, suggesting that ubiquitin is not required for this process.^161^ In addition, independently of its LC3 binding ability, PHB2 stabilizes PINK1 on damaged mitochondria by negatively modulating PARL activity.^162^ In *C. elegans*, PHB2 is essential for paternal mitochondrial elimination.^161^ The pleiotropic role played by PHB2 in mitophagy warrants further investigation.

#### Lipids as mitophagy receptors

Emerging evidence suggests that specific lipids, such as cardiolipin (CL) and ceramides, can function as mitophagy receptors upon mitochondrial damage.

##### Cardiolipin

CL is a unique phospholipid localized primarily to the IMM and to contact sites between the IMM and the OMM, constituting ~15% of IMM phospholipid mass.^163,164^ Whereas other phospholipids consist of a glycerol backbone, a polar head group, and two hydrophobic acyl chains, CL has a unique dimeric structure composed of two glycerol moieties linked by a third and bearing four acyl chains. This structure enables CL to interact with IMM proteins involved in the ETC and maintain the organization of mitochondrial cristae.^165^ In healthy mitochondria, most CL is localized on the IMM, where it affects diverse cellular processes^166^; upon mitochondrial damage, it translocates to the OMM, increasing its local concentration on the OMM.^167^ This redistribution enables CL to access and bind LC3, particularly LC3A, facilitating mitophagy.^167,168^

Disruption of CL function — whether by blocking its synthesis via CL synthase knockdown, inhibiting its translocation to the OMM via phospholipid scramblase-3 knockdown,^169^ or interrupting its remodeling via knockdown of the phospholipid transacylase tafazzin^170^ — impairs mitophagy.

CL also engages other effector proteins to facilitate mitophagy. Beclin 1, for example, has an evolutionarily conserved domain with high affinity for CL-enriched membranes and binds CL via its aromatic finger, inducing deformation of membranes and liposomes.^171^ Proteins related to mitochondrial dynamics, such as OPA1^172^ and DRP1,^173,174^ also interact with CL, which promotes their oligomerization and functional activation. The intermembrane-space-protein nucleoside diphosphate kinase D (NDPK-D, also known as NM23-H4) binds CL and facilitates its redistribution to the OMM.^175,176^ In addition, NDPK-D also forms a complex with OPA1, a process closely associated with its mitophagy-inducing CL-transfer activity.^176^ Collectively, these findings suggest that fission–fusion dynamics are involved in the regulation of mitophagy.

##### Ceramides

Ceramides are central molecules of sphingolipid metabolism; they are composed of a sphingosine base linked to an acyl chain that varies in length from C14 to C26.^177^ Their subcellular localization and acyl chain length are important for cellular function. Expression of ceramide synthase 1 (CerS1) promotes the production of C18-ceramide, which mediates LC3B lipidation and LC3B-II formation. C18-ceramide subsequently interacts with LC3B-II, directing LC3B-II-containing autophagosomes to the mitochondria to initiate mitophagy, ultimately inhibiting mitochondrial function and reducing oxygen consumption.^178^ This interaction is regulated by DRP1, and depletion of DRP1 alters the mitochondrial localization of ceramides and prevents the mitochondrial targeting of autophagosomes.^178^ Upon sodium selenite treatment, mitochondrial membrane rearrangements drive recruitment of the p17/PERMIT-CerS1 complex, and loss of p17/PERMIT reduces CerS1 translocation and ceramide-mediated mitophagy both in vitro and in vivo. Notably, p17/PERMIT knockout mice demonstrate aging-dependent sensorimotor deficiency that can be mitigated by administration of a ceramide analog.^179^ This aligns with findings that CerS1 inhibition exacerbates age-related skeletal muscle dysfunction.^180^ Activating this pathway with sodium selenite further improved motor-neuron deficits in aged wild-type mice.^179^ The outcome of modulating ceramide-mediated mitophagy varies depending on the cellular and physiological context. For example, in multiple human head and neck squamous cell carcinoma (HNSCC) cell lines, C18-ceramide-mediated mitophagy triggers caspase-independent autophagic cell death, which leads to tumor-suppressive effects in vivo.^178^ Furthermore, ceramide-mediated mitophagy is upregulated upon PINK1 deficiency, suggesting a compensatory mechanism for maintenance of mitochondrial quality control.^181^

### Piecemeal mitophagy

In addition to wholesale mitophagy, a specialized form of mitophagy known as piecemeal mitophagy selectively engulfs and degrades individual mitochondrial proteins or protein complexes. Unlike bulk mitophagy, piecemeal mitophagy occurs independently of mitochondrial depolarization and Parkin overexpression, functioning as a basal quality-control mechanism essential for maintenance of a healthy mitochondrial network. Certain mitochondrial proteins, e.g., SAMM50 and metaxin 1 (MTX1) from the sorting and assembly machinery (SAM) complex and mitochondrial inner membrane protein (IMMT, also known as MIC60) from the mitochondrial contact site and cristae organizing system (MICOS) complex, become autophagy cargo by interacting with ATG8 family proteins, facilitating their autophagosomal degradation in a p62-dependent manner.^182,183^

Under hypoxia-mimicking conditions, PX-domain-containing protein sorting nexin 10 (SNX10) translocates from early endosomal compartments to late endosomal structures containing piecemeal mitophagy markers. SNX10 acts as a negative regulator of the piecemeal mitophagy of OXPHOS machinery components, and its depletion leads to enhanced degradation of specific mitochondrial proteins, reduced OXPHOS activity, and increased ROS accumulation.^184^ This suggests that piecemeal mitophagy plays a crucial role in rewiring cellular metabolism toward OXPHOS. The dual localization of SNX10 under normal and hypoxia-mimicking conditions further implies that interorganellar communication is essential for the coordination of mitophagy quality control.

Notably, piecemeal mitophagy extends beyond proteins to include damaged mtDNA. mtDNA is particularly susceptible to damage under oxidative stress, and under such conditions, the primate-specific mitophagy receptor ATPase family AAA domain-containing protein 3B (ATAD3B) mediates piecemeal mitophagy to selectively remove damaged mtDNA.^185^ Mechanistically, ATAD3B normally hetero-oligomerizes with ATAD3A, localizing its C-terminus to the mitochondrial intermembrane space. mtDNA damage or depletion disrupts the complex, exposing the ATAD3B C-terminus on the OMM to recruit LC3 and trigger mitophagy. This mechanism aligns with the characteristics of piecemeal mitophagy, which targets discrete mitochondrial components rather than entire organelles (Box 3).

Box 3 Mitochondria-derived vesicles In addition to mitophagy, mitochondria can also shed vesicles containing mitochondrial components for degradation to facilitate mitochondrial quality control. Such mechanisms include MDVs,^19,20^ SPOTs,^21^ and VDIMs,^22^ among which MDVs are the best characterized.^19^ MDVs are mitochondria-derived single- or double-membraned structures with sizes of 60–150 nm. Under basal and stressed conditions, different populations of MDVs carry selected mitochondrial components to multivesicular bodies/lysosomes for degradation.^19,20,475,476^ For example, steady-state MDVs contain the TOM complex, and their formation is initiated by MIRO1/2-dependent thin membrane protrusions and requires DRP1-mediated scission.^476^ By contrast, TOM20-negative MDVs form upon oxidative stress induced by antimycin A and deliver selected components of the mitochondrial matrix and IMM to lysosomes.^477^ This process requires both PINK1 and Parkin for its generation and involves syntaxin-17-mediated fusion with lysosomes.^477,478^ The MDV pathway appears to fine-tune the mitochondrial proteome upon mild mitochondrial stress, as it enables the timely removal of damaged mitochondrial components. However, when mitochondrial damage exceeds a certain threshold, mitophagy is activated to degrade the entire organelle. Consistent with this scenario, rare surviving autophagy-deficient cancer cells adapt to the loss of autophagy/mitophagy, possibly through increased mitochondrial fusion and MDV formation.^479^ In addition, loss of USP30 has been shown to increase TOM^+^ MDVs.^476^

## Mitophagy in disease

**Fig. 3:**
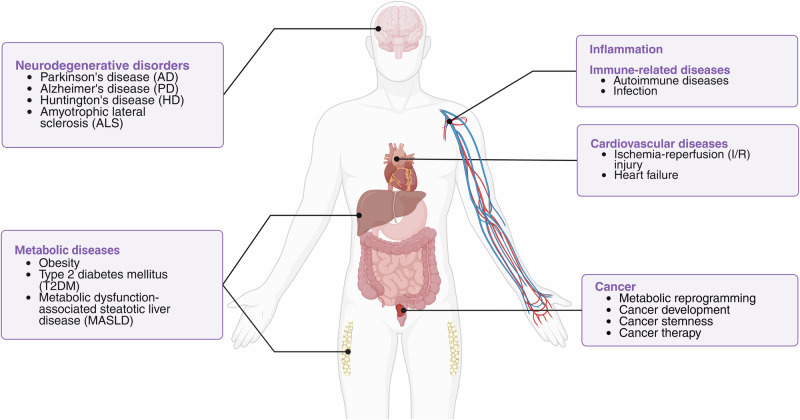
Summary of mitophagy-associated human diseases. This review discusses the mitophagy dysregulation underlying neurodegenerative, cardiovascular, metabolic, and immune-related disorders, as well as cancer.

Dysregulated mitophagy is increasingly recognized as a contributor to the pathogenesis of diverse diseases, including neurodegenerative, cardiovascular, and metabolic disorders, as well as autoimmune diseases and cancer. Different organ systems display various levels of tolerance to mitophagy deficiency. Tissues differ in their metabolic demands, and those with greater reliance on mitochondrial function are more vulnerable to the consequences of mitochondrial decline; mitochondrial quality control relies on multiple mechanisms, including mitophagy, mitochondrial dynamics, biogenesis, and stress-response pathways.^186^ Organs or cell populations often differ in their preferred pathways, leading to heterogeneous susceptibility to mitophagy deficiency. For instance, mitochondrial protein turnover is reported to be faster in the heart than in the brain.^187^ Moreover, even within the same organ, mitophagy levels vary across regions or cell types. For example, the dentate gyrus exhibits enriched mitophagy, whereas the substantia nigra shows modest levels; the Purkinje cell layer in the cerebellum displays high levels of mitophagy.^89^ On the other hand, enhancing mitophagy can also lead to undesirable outcomes under certain circumstances, such as during acute infection or cancer development. This section will explore the roles of mitophagy in specific diseases (Fig. 3), highlighting key findings from recent research and underscoring the physiological relevance of the diverse mitophagy pathways.

### Neurodegenerative diseases

Neurodegenerative diseases represent a heterogeneous group of disorders characterized by progressive neuronal loss and functional decline in the central nervous system (CNS). Emerging evidence underscores the pivotal role of mitophagy in the pathogenesis of conditions such as PD, Alzheimer’s disease (AD), Huntington’s disease (HD), and amyotrophic lateral sclerosis (ALS). Dysregulation of mitophagy disrupts mitochondrial homeostasis, leading to energy deficits, dysregulated signal transmission, oxidative stress, neuroinflammation, and apoptotic signaling, which collectively drive neurodegeneration.^188–190^

#### Parkinson’s disease

PD is a progressive neurodegenerative disorder characterized by the loss of dopaminergic neurons in the substantia nigra, leading to hallmark motor symptoms such as tremors, rigidity, and bradykinesia. Mitophagy impairment is a key pathological feature observed in PD patient tissues and experimental models. Mutations in genes encoding PINK1 and Parkin are associated with early-onset PD,^25,26,190–192^ and human genetic analyses have implicated multiple additional genes in PD pathogenesis.^193–195^ Pathogenic variants in key regulators of the PINK1–Parkin mitophagy pathway lead to defective mitochondrial clearance, resulting in accumulation of dysfunctional mitochondria, exacerbated oxidative stress, and bioenergetic deficits — hallmarks of dopaminergic neuron degeneration.

Pathogenic mutations in the *PRKN* gene, ranging from point substitutions to large deletions, often result in loss of enzymatic activity, impaired substrate ubiquitination, and protein destabilization, which collectively disrupt proteasomal degradation pathways.^196–198^ In parallel, α-synuclein (αSyn), a protein prone to aggregation in PD, exacerbates mitochondrial damage by disrupting PINK1 stabilization and Parkin recruitment.^199,200^ The mutant αSyn variant A53T activates p38 MAPK, which phosphorylates Parkin at Ser131, impairing its E3 ligase activity and promoting neuronal death.^201^ *Drosophila* models further confirm that loss of PINK1–Parkin leads to mitochondrial fragmentation, locomotor defects, and dopaminergic neuron loss, underscoring the evolutionary conservation of this pathway.^202,203^

Similar to mutations in *PINK1* and *PRKN*, mutations in other mitophagy-related genes also contribute to PD pathogenesis. For example, the leucine-rich repeat kinase 2 (*LRRK2*) G2019S mutation delays mitochondrial arrest and promotes PD onset by preventing MIRO1 removal from the OMM, thereby reducing OPTN-dependent mitophagy.^204,205^ Promoting MIRO1 degradation enhances clearance of damaged mitochondria and protects PD neurons against mitochondrial stress^206^ (related to Box 4). Intriguingly, LRRK2 inhibitors restore mitophagic flux independently of the PINK1–Parkin pathway, suggesting alternative mechanisms for therapeutic targeting.^207^ These findings highlight the complexity of mitophagy regulation and its intersection with PD pathology.

#### Alzheimer’s disease

AD is the most prevalent neurodegenerative disease, characterized by amyloid-β (Aβ) plaques and hyperphosphorylated Tau (pTau).^208^ Mitochondrial dysfunction and oxidative stress are early events in AD pathogenesis.^209^ Functional mitochondria help to reduce aberrant amyloid precursor protein (APP) processing and prevent excessive Aβ accumulation. Recent evidence indicates that mitophagy is impaired in the hippocampus of AD patients, human AD neurons derived from induced pluripotent stem cells (iPSCs), and AD animal models.^210^ Moreover, mitophagy defects are strongly correlated with AD progression.^211–213^ In animal models of AD, impaired mitophagy is associated with reduced cytosolic Parkin levels and aberrant PINK1 accumulation, exacerbating Aβ-induced toxicity.^214^ Consistent with these findings, restoration of Parkin expression mitigates mitochondrial deficits, reduces Aβ burden, lowers neuroinflammation, and ameliorates cognitive decline.^210,215^

Tau pathology further complicates mitochondrial dynamics. On one hand, N-terminal tau fragments have been reported to sequester Parkin and ubiquitin C-terminal hydrolase L1 (UCHL1), redirecting them to mitochondria and triggering excessive organelle clearance.^216^ On the other hand, full-length tau inhibits Parkin translocation by elevating mitochondrial membrane potential or by directly binding Parkin in the cytosol.^217^ This dual role of tau — acting as both a mitophagy inducer and suppressor — reflects its stage-dependent effect on neuronal survival. Notably, enhancement of mitophagy through pharmacological and genetic interventions reduces AD-related tau hyperphosphorylation in human neuronal cells and restores memory function in transgenic tau nematodes and mice.^210^

#### Huntington’s Disease

HD is an inherited neurodegenerative disorder typically caused by a mutation in the gene encoding huntingtin (HTT), which leads to the accumulation of mutant huntingtin (mHTT) protein with polyglutamine (polyQ) repeats at the N terminus.^218^ HD is characterized by motor dysfunction, cognitive decline, and psychiatric symptoms such as depression and irritability. HD patients exhibit fragmented mitochondria and reduced mitochondrial mass. Moreover, mHTT severely impairs ATP production and respiration rate, suggesting that dysregulated energy metabolism may be a key feature of HD.^219^ Mounting evidence indicates that impaired mitochondrial clearance in HD reflects dysregulation of mitophagic pathways.^220,221^ mHTT shows reduced binding affinity with the selective autophagy receptor p62^220^ and impaired ULK1/PI3KC3 complex formation, hindering autophagosome–lysosome fusion.^221^ Consequently, damaged mitochondria accumulate, amplifying ROS production and caspase activation. PINK1 overexpression partially restores mitophagy in HD models, indicating that compensatory pathways may bypass some mHTT-induced defects.^222^ Interestingly, regional analysis of *Pink1*-deficient models reveals striking neuroanatomical heterogeneity in mitophagic activity,^223^ implying that differences in neuronal susceptibility may stem from distinct reliance on Parkin-dependent versus alternative mitochondrial clearance mechanisms under varying physiological conditions. Thus, precise modulation of mitophagy is critical for maintaining redox balance and cellular homeostasis in HD.

#### Amyotrophic Lateral Sclerosis

ALS is a motor neuron disease, a progressive and ultimately fatal neurodegenerative disorder characterized by motor neuron degeneration and subsequent muscle atrophy. A key pathological feature of ALS is the presence of abnormal protein aggregates, known as inclusion bodies, in the cytoplasm of neurons, which contribute to a vicious cycle that exacerbates oxidative damage.^224,225^ Mitochondrial dysfunction is a hallmark of ALS, and emerging evidence suggests that impaired mitophagy plays a critical role in disease progression. Several ALS-associated genes, including *C9ORF72*,^226^ superoxide dismutase 1 (*SOD1*),^227^ *OPTN*,^228,229^ *TBK1*,^229,230^ and TAR DNA-binding protein 43 (*TDP-43*),^231^ are directly implicated in the regulation of mitophagy. Mutations in these genes typically disrupt mitochondrial quality control, leading to the accumulation of damaged mitochondria and neurotoxic protein aggregates that are key pathological features of ALS. For instance, *C9ORF72* mutations, the most common genetic cause of ALS, are strongly linked to mitochondrial dysfunction. Its pathogenic GGGGCC repeat expansion leads to the production of toxic dipeptide repeat proteins, which interact with mitochondria, disrupt membrane potential, and elevate ROS levels, ultimately impairing mitophagy.^226^ Similarly, mutant SOD1 aggregates within the mitochondrial intermembrane space, disrupts mitochondrial retrograde transport, and sequesters OPTN, suppressing mitophagosome formation.^227^ However, *Prkn* knockout in *SOD1* G93A models unexpectedly mitigates mitochondrial depletion, delaying ALS progression and prolonging survival.^232^ These contrasting outcomes suggest that mitophagy requirements in ALS may be cell type- and mutation-specific, emphasizing the need for personalized therapeutic strategies.

Box 4 Mitochondrial dynamics and mitophagy Mitochondrial fusion, fission, and transport are collectively referred to as mitochondrial dynamics. Disruption of these processes has been linked to numerous human diseases.^480–482^ Mitochondrial dynamics is tightly coupled with mitophagy to maintain a healthy mitochondrial network.

Mitochondrial fusion joins two mitochondria at the OMM and IMM, a process mediated by three membrane GTPases, namely MFN1, MFN2,^483,484^ and OPA1.^485^ MFN1 and MFN2 are primarily responsible for OMM tethering and fusion, whereas OPA1 mediates IMM fusion. Mitochondrial fission is the division of a single mitochondrion into two mitochondria, mainly orchestrated by DRP1^486^ and OMM-located receptors such as the mitochondrial fission factor (MFF), mitochondrial dynamics proteins 49 and 51 (MID49/51), and mitochondrial fission protein 1 (FIS1).^487^ Fission occurs primarily at mitochondria–endoplasmic reticulum (ER) contact sites (MERCS),^488^ where the ER and actin coordinate to drive initial mitochondrial constrictions.^487^ Recruited DRP1 forms a ring-like structure around the mitochondrion to drive scission.^486^

Fusion enables mitochondria to exchange intramitochondrial components for optimal function, whereas fission is required for the generation of new mitochondria^489^ and enables the segregation of damaged mitochondrial components for disposal.^490^ Mitochondria go through frequent cycles of fusion and fission in a ‘kiss and run’ pattern, which coordinates with mitophagy to ensure mitochondrial homeostasis. For example, during starvation, mitochondria become elongated and interconnected and are spared from autophagic degradation to preserve ATP production.^491,492^ Asymmetric fission can lead to the creation of unequal daughter organelles, with one daughter mitochondrion showing increased membrane potential and a high probability of fusion, whereas the other has decreased membrane potential and is preferentially targeted by mitophagy.^490,493^ Furthermore, the proteins that control mitochondrial dynamics can directly interact with those that regulate mitophagy. For example, in PINK1–Parkin-mediated mitophagy, MFN2 mediates Parkin recruitment to damaged mitochondria in a PINK1-dependent manner.^494^ Recruited Parkin then ubiquitinates MFN1/2 for proteasomal degradation,^61,495^ enabling dissociation of mitochondria from the ER^496,497^ to prevent refusion and drive mitophagy. The engagement of mitochondrial dynamics in other mitophagy pathways is discussed further in the receptor-mediated mitophagy section.

The transport of mitochondria is essential to meet local demands. To transport mitochondria as cargo, MIRO1/2^498^ recruit trafficking kinesin-binding protein 1/2 (TRAK1/2) adaptor proteins,^499,500^ which in turn recruit motor proteins, namely kinesin and dynein-dynactin, to transport mitochondria toward the plus- and minus-ends of microtubules, respectively. Mitochondrial trafficking is especially critical in neurons, as mitochondria must travel long distances along microtubules to axons, synapses, and dendrites — a process that could take days.^501^ It is plausible that mitophagy and transport cooperate to ensure adequate ATP supply and calcium buffering. Several lines of evidence support this idea. (i) Upon mitochondrial depolarization, activation of the PINK1–Parkin pathway triggers rapid degradation of MIRO1/2, releasing kinesin from mitochondria and thereby arresting mitochondrial movement.^502^ (ii) Deficiency of MIRO1/2 suppresses mitophagy.^503^ (iii) *Pink1* mRNA is cotransported with mitochondria to neurites, enabling local translation of the short-lived PINK1 and ensuring that the PINK1–Parkin pathway can effectively monitor mitochondrial health even at sites distal to the soma.^504^ Furthermore, recent evidence suggests that aberrant MIRO accumulation is associated with PD, and promoting MIRO1 degradation can rescue dopaminergic neurodegeneration.^204,206^ Deciphering the crosstalk between mitochondrial transport and mitophagy may unlock novel therapeutic strategies for neurodegenerative disorders.^505^

### Inflammation and Immune-related Diseases

Mitophagy has emerged as a linchpin connecting mitochondrial integrity to immune homeostasis. By regulating inflammasome activity, interferon production, chemotaxis, lymphocyte metabolism, and pathogen responses, it ensures balanced immune activation while preventing chronic inflammation. Dysregulation of mitophagy is closely linked to the pathogenesis of various immune-related disorders.^5^

#### Inflammation

Many mitochondrial components share similar structures with those of their bacterial ancestor. These components, such as mtDNA, mitochondrial RNA (mtRNA), mitochondrial ROS (mtROS), and N-formyl peptides, can be released upon mitochondrial stress or damage. They function as endogenous damage-associated molecular patterns (DAMPs) and are recognized by pattern recognition receptors (PRRs) to initiate inflammatory responses.^5,233–236^

mtDNA is among the most widely investigated mitochondrion-derived DAMPs^5,233–235,237^ and resembles bacterial DNA in being circular and having unmethylated cytidine-phosphate-guanosine (CpG) motifs. Upon release into the cytosol, mtDNA can activate the cyclic GMP–AMP synthase (cGAS)–stimulator of interferon genes (STING) pathway to promote type I interferon (IFN) production.^238–241^ It can also activate the absent in melanoma 2 (AIM2) inflammasome.^242^ Furthermore, mtDNA can activate the NOD-, LRR-, and pyrin domain-containing protein 3 (NLRP3) inflammasome in a ROS-dependent manner,^243–245^ leading to caspase-1-dependent secretion of interleukin-1β (IL-1β) and IL-18 cytokines. Moreover, circulating or cytosolic mtDNA can also activate Toll-like receptor 9 (TLR9)-mediated immune responses.^246–248^

Double-stranded mtRNA can also be recognized by cytosolic RNA virus receptors, including retinoic acid-inducible gene I (RIG-I)^249^ and melanoma differentiation-associated protein 5 (MDA5),^250^ to activate mitochondrial antiviral signaling protein (MAVS) and induce type I IFN expression. Mitochondria-derived N-formyl peptides can activate formyl-peptide receptors (FPRs),^247,251^ and mtROS further contribute to the inflammatory responses of immune cells.^252,253^

Mitophagy removes dysfunctional mitochondria, thereby preventing the release of mitochondrial components into the cytosol or extracellular space and reducing inflammation. Consistent with this notion, the depletion of autophagic proteins, such as LC3B and Beclin 1, causes accumulation of dysfunctional mitochondria and cytosolic release of mtDNA, leading to enhanced secretion of IL-1β and IL-18 in macrophages.^244^ Upon pressure overload, lysosomal deoxyribonuclease (DNase) II deficiency in cardiomyocytes leads to mtDNA accumulation in autolysosomes, which subsequently activates TLR9 signaling-mediated inflammatory responses, contributing to heart failure.^254^ Accordingly, enhancement of mitophagy can put the brakes on inflammatory responses. For example, nuclear factor κB (NF-κB), a key activator of the inflammatory response, restrains its own inflammation-promoting activity by inducing p62-dependent mitophagy, which in turn inhibits NLRP3 inflammasome activity. Macrophages lacking p62, Parkin, or ATG7 display pronounced inflammasome activation.^255^ A combination of Pink1 overexpression and Torin-1 treatment restores mitophagic flux in aged macrophages, thereby inhibiting cGAS–STING activation.^256^

Intriguingly, mitophagy contributes to immune regulation beyond the control of mitochondrial DAMPs. (i) Mitophagy is involved in metabolic reprogramming during immune cell activation. For instance, macrophages stimulated by proinflammatory factors such as lipopolysaccharide (LPS) and IFNγ have lower mitochondrial mass than those stimulated by the anti-inflammatory cytokines IL-4/IL-13. Inhibition of autophagy/mitophagy reduces the proinflammatory activation of macrophages by limiting the glycolytic switch in response to proinflammatory stimuli.^105^ In line with this, IL-10 exerts its anti-inflammatory effect by counteracting LPS-induced metabolic reprogramming of macrophages, a process that involves mitophagy induction to limit the accumulation of dysfunctional mitochondria and mtROS.^257^ (ii) Mitophagy and inflammation share several common regulators, with TBK1 being a prominent example. As mentioned above, TBK1 plays important roles in Parkin-mediated mitophagy and innate immunity. Upon ligand binding, PRRs such as RIG-I, cGAS, and TLR3/4 activate the downstream immune adaptor proteins MAVS, STING, and TIR domain-containing adapter-inducing interferon-β (TRIF), respectively. Activated adaptor proteins then recruit and activate TBK1, as evidenced by the activating phosphorylation of TBK1 at Ser172, which in turn phosphorylates the immune adaptor proteins and interferon regulatory factor 3 (IRF3) to trigger type I IFN responses.^258^ During mitophagy, mitochondrial depolarization leads to TBK1 phosphorylation at Ser172, and TBK1 subsequently recruits selective autophagy receptors, including OPTN and NDP52.^74^ Interestingly, although TBK1 activation upon LPS stimulation leads to OPTN phosphorylation,^65^ its activation upon mitochondrial depolarization does not activate IRF3.^74^ How this functional compartmentalization of TBK1 is achieved remains to be explored. The immune receptor protein MAVS provides another link, as mitochondria provide a scaffold for MAVS, and activated MAVS can induce mitophagy.^259^ These findings add further complexity to the role of mitophagy in inflammation, and a clearer picture of its diverse functions is only beginning to emerge.

Nonetheless, it is important to bear in mind that inflammation acts as a double-edged sword. Although an appropriate host inflammatory response is crucial for controlling infection, dysregulated inflammation can lead to organ failure and is a common feature of many diseases. Thus, although enhancing mitophagy generally reduces inflammation, its effects are highly dependent on context. For example, the release of mtDNA plays a crucial role in immune activation, as it fuels cGAS–STING signaling in response to intracellular pathogens such as herpesvirus,^241^ influenza virus,^260^ dengue virus,^261^ and severe acute respiratory syndrome coronavirus 2 (SARS-CoV-2),^262^ as well as extracellular bacteria such as *Pseudomonas aeruginosa*.^263^ In such cases, mitophagy limits the release of mtDNA and may dampen protective immune responses against pathogens. By contrast, in aged mice, pharmacological induction of mitophagy attenuates cGAS–STING activation and ameliorates age-associated neurological decline.^264^ Further examples are discussed in the following sections on autoimmune diseases and viral or bacterial infections.

#### Autoimmune diseases

Aberrant mitophagy has been implicated in autoimmune diseases such as systemic lupus erythematosus (SLE) and inflammatory bowel disease (IBD).^4^ Impaired mitophagy contributes to mitochondrial dysfunction in lupus T cells, leading to necrosis and the release of immunogenic debris that triggers autoantibody production and type I IFN responses.^265^ In addition, SLE neutrophils exhibit defective mitophagy, resulting in the retention of oxidized mtDNA, which activates dendritic cells and promotes pathogenic autoimmunity via AIM2 inflammasome activation and TLR9 signaling.^266^ Patients with SLE exhibit reduced expression of mitophagy-related genes, which correlates with disease severity.^267^

Moreover, genetic variants of mitophagy-related genes, such as *ATG16L1* and immunity-related GTPase M (*IRGM*), disrupt mitochondrial quality control in IBD. The *ATG16L1* T300A variant impairs mitophagic flux, resulting in ROS accumulation, NLRP3 inflammasome activation, and elevated IL-1β secretion in macrophages.^268^ Likewise, IRGM deficiency exacerbates mitochondrial dysfunction, causing Paneth cell abnormalities and impaired bacterial clearance, which are hallmarks of Crohn’s disease.^269^

#### Viral/bacterial infection

Mitophagy plays a key role in the host response to infections. As a case in point, LPS and IFNγ can inhibit PINK1-dependent mitophagy in macrophages, which in turn triggers macrophage activation in an mtROS-dependent manner.^270^ In a mouse model of polymicrobial infection, transfer of *Pink1*-deficient bone marrow or pharmacological inhibition of mitophagy improved bactericidal clearance and increased survival rate, whereas the promotion of mitophagy led to increased bacterial load and lower survival rate.^270^ Consistent with these findings, *Prkn* knockout mice exhibit enhanced activation of the NLRP3 inflammasome by ROS, elevated innate antiviral inflammation, and increased viral clearance.^271^ In addition, Parkin expression was reduced in peripheral blood mononuclear cells from virally infected patients,^271^ while, in critically ill cases, patients with sepsis showed inhibited mitophagy in their blood monocytes compared with non-septic patients, suggesting its potential utility as a biomarker for sepsis diagnosis.^270^

However, some pathogens can exploit mitophagy to promote their survival. Viral proteins, such as the matrix protein of human parainfluenza virus 3 (HPIV3) and PB1-F2 of influenza A virus (IAV), directly interact with autophagy-related proteins like ATG8 and mitochondrial Tu translation elongation factor (TUFM) to induce ubiquitin-independent mitophagy. This process disrupts MAVS-dependent interferon production, thereby dampening antiviral responses.^272–274^ Similarly, SARS-CoV-2 open reading frame 10 (ORF10) bridges NIX and LC3B to activate mitophagy, impairing MAVS signaling and promoting viral persistence.^275^

### Cancer

Cancer is characterized by uncontrolled cell proliferation and survival, often driven by metabolic reprogramming and mitochondrial dysfunction. Emerging evidence highlights the dual roles of mitophagy in cancer biology: mitophagy can function as either a tumor suppressor or a promoter depending on context-specific factors such as cancer type, stage, and genetic background.^95^

#### Metabolic Reprogramming

Cancer cells often exhibit metabolic reprogramming, favoring glycolysis over OXPHOS even under aerobic conditions, a phenomenon known as the Warburg effect. Mitophagy contributes to this metabolic shift by reducing mitochondrial mass and suppressing OXPHOS. In *KRAS* mutant pancreatic cancer cells, elevated NIX-mediated mitophagy diminishes mitochondrial networks, thereby enhancing glycolytic flux to meet the bioenergetic demands of rapid cell proliferation.^276^ Parkin orchestrates glycolytic regulation through diverse molecular pathways. For example, it binds directly to and suppresses the enzymatic activity of pyruvate kinase M2 (PKM2), thereby regulating the glycolytic pathway in glioblastoma and lung cancer cells.^277^ Parkin also affects cancer metabolism by targeting HIF-1α for proteasomal degradation, leading to attenuation of breast cancer metastasis.^278^ Genetic ablation of *Prkn* disrupts cellular homeostasis through PTEN destabilization, resulting in constitutive activation of the PI3K/AKT pathway and promotion of tumorigenesis.^279^ In addition to these direct interactions, mitophagy participates in metabolic reprogramming by selectively degrading hexokinase 2 (HK2), thereby coupling mitochondrial surveillance to glycolytic enzyme turnover.^280^

#### Cancer Development

The regulatory role of mitophagy in cancer progression exhibits significant tissue-specific heterogeneity. Parkin is frequently deleted or mutated in cancers such as glioblastoma, breast cancer, colorectal cancer, and ovarian cancer.^281–284^ Experimental models have revealed that Parkin-knockout mice are highly susceptible to spontaneous hepatocellular carcinoma (HCC).^285^ Loss of Parkin promotes tumorigenesis by disrupting mitophagy, leading to ROS accumulation, genomic instability, and resistance to apoptosis. Consistent with these findings, pharmacological activation of PINK1–Parkin-mediated mitophagy inhibits the growth of HCC,^286^ colorectal cancer,^287^ and pancreatic cancer.^288^ By contrast, Parkin exhibits an oncogenic function in melanoma models, as its genetic ablation inhibits tumor growth and metastasis by destabilizing MFN2.^289^

Some mitophagy-related proteins also exhibit dual oncogenic and tumor-suppressive functions. For instance, loss of PINK1 in glioblastoma stabilizes HIF-1α, stimulating the Warburg effect and tumor proliferation,^290^ whereas *PINK1* silencing blocks the cell cycle and promotes apoptosis of lung cancer cells.^291^ The subcellular localization of BNIP3 significantly influences its function in cancer progression. Nuclear BNIP3 in non-small cell lung cancer is associated with poor survival,^292^ whereas cytoplasmic localization of BNIP3 in breast cancer may suppress malignancy by modulating ROS levels.^293^ FUNDC1, another hypoxia-induced mitophagy receptor, also plays a dual role in modulating cancer development and progression. In early-stage cervical cancer, FUNDC1 expression is significantly elevated in tumor cells compared with normal tissues, and its high expression is associated with poor patient prognosis. Inhibition of FUNDC1 enhances the sensitivity of cancer cells to ionizing radiation and cisplatin, suggesting its involvement in therapeutic resistance.^294^ Conversely, in hepatocytes, FUNDC1 silencing leads to the accumulation of dysfunctional mitochondria, release of mtDNA, overactivation of caspase-1, and excessive IL-1β production, thus promoting the initiation and progression of HCC.^295^ These opposing outcomes emphasize the need to evaluate the context-dependent role of mitophagy in cancer development.

#### Cancer stemness

Cancer stem cells (CSCs) are a subpopulation with self-renewal capacity that rely on mitophagy to preserve their stem-like properties. In hematopoietic stem cells, PINK1/Parkin-mediated mitophagy preserves self-renewal capacity, whereas its disruption impairs stem cell function.^296^ Consistent with these observations, hepatic CSCs use mitophagy to degrade the tumor suppressor p53, preventing its nuclear translocation and thus evading apoptosis, which is critical for stemness maintenance.^297^ Mitochondrial dynamics also modulate CSC behavior through coupling with mitophagy. For instance, FIS1 is required for the activation of mitophagy in leukemia stem cells, which sustains their self-renewal capacity and inhibits differentiation.^298^ Moreover, mitochondrial fission factor is significantly upregulated in liver cancer-initiating cells, enhancing mitochondrial fission and directing healthy mitochondria to daughter CSCs while targeting damaged ones for degradation by mitophagy, thereby promoting tumor-initiating potential.^299^

#### Cancer Therapy

Mitophagy affects cancer therapy through pleiotropic mechanisms involving mtDNA release, immune signaling pathways, and crosstalk with treatments like chemotherapy, radiation, and immunotherapy.

Chemotherapeutic agents often induce mitochondrial damage in cancer cells, triggering mitophagy as a protective mechanism that promotes cancer cell survival and drives drug resistance.^300,301^ As mentioned previously, ARIH1-mediated mitophagy enhances chemoresistance in breast and lung adenocarcinomas by supporting tumor cell survival.^300^ Moreover, tumor cells express programmed death-ligand 1 (PD-L1) to engage with PD-1 on T cells, thereby escaping antitumor immunity. Paclitaxel, a widely used chemotherapeutic agent, increases ATAD3A expression and thereby prevents PINK1-dependent mitophagy, which impairs the degradation of PD-L1 and contributes to therapy resistance.^301^

On the other hand, mitophagy affects anti-tumor immunity via the cGAS–STING pathway. For example, autophagy deficiency increases the efficacy of radiotherapy in breast cancer by promoting mtDNA release, which activates the cGAS–STING pathway to induce type I IFN secretion.^302^ Similarly, pharmacological inhibition of caspase-9 with emricasan restores tumor-intrinsic mtDNA sensing, and its combination with radiation and anti-PD-L1 therapy improves therapeutic efficacy in colon adenocarcinoma cells.^303^ Furthermore, the BCL-2 inhibitor ABT-199 induces VDAC1 oligomerization to promote mtDNA release, thereby activating STING signaling to enhance chemokine expression and cytotoxic T cell infiltration. In vivo studies have shown that ABT-199 significantly improves the antitumor efficacy of anti-PD-L1 therapy.^304^

Mitophagy also plays a pivotal role in optimizing antitumor T cell therapy. A major challenge in cancer immunotherapy is the exhaustion of tumor-infiltrating T cells, and recent studies suggest that the modulation of mitophagy could mitigate this effect. Specifically, PINK1-mediated mitophagy triggered by urolithin A (UA) promotes the expansion of T memory stem cells (T_SCM_), ultimately enhancing the efficacy of immunotherapy in colorectal cancer.^287^ In addition, genetic or pharmacological inhibition of USP30 restores mitophagy, rejuvenates exhausted T cell function, and suppresses tumor growth in murine colon adenocarcinoma.^305^

Although cancer therapy is often focused on the elimination of tumor cells, minimizing treatment-induced side effects is equally important, and mitophagy affects this aspect as well. Bax/Bak-mediated apoptosis activates AMPK/ULK1-dependent mitophagy to clear damaged mitochondria. This mitophagic activity suppresses the secretion of IFN-β, thereby maintaining immunological silence during cell death. This mechanism is critical for preventing inflammation-driven complications in cancer therapy.^306^

Overall, mitophagy exerts multifaceted effects in cancer therapy. It can promote cancer cell survival and therapy resistance while also enhancing antitumor immunity, either by triggering mtDNA release to activate the cGAS–STING pathway or by optimizing T cell therapy. Future strategies should aim to dynamically fine-tune mitophagy on the basis of immune context (activation or suppression) and to develop combinations targeting ATAD3A or PINK1 pathways.

### Metabolic Diseases

Metabolic diseases, including obesity, type 2 diabetes, and metabolic dysfunction-associated steatotic liver disease (MASLD), are characterized by dysregulation of glucose and lipid metabolism. Mitochondrial dysfunction and impaired mitophagy contribute to the pathogenesis of these diseases.

#### Obesity

Obesity, characterized by excessive fat accumulation and adipocyte hypertrophy, is closely associated with mitochondrial dysfunction, which contributes to metabolic disturbances such as insulin resistance, chronic inflammation, and oxidative stress.^307^ The interplay between mitophagy and obesity is complex, with mitophagy acting as both a protective and detrimental mechanism, depending on the context and extent of its activation.^308^ For example, global or brown adipocyte-specific deletion of *Pink1* induces brown adipose tissue dysfunction and an obesity-prone phenotype in mice.^309^ Consistent with these findings, mice with a deficiency in FUNDC1 exhibit defective mitophagy and develop more severe obesity and insulin resistance when fed a high-fat diet (HFD).^310^ However, excessive mitophagy may also have detrimental effects on lipid metabolism. For example, adipose-specific deletion of the mitochondrial redox gene *Trx2* induces excessive mitophagy, resulting in reduced mitochondrial mass and impaired energy production in white adipose tissue. This, in turn, increases lipolysis, exacerbates metabolic dysfunction, and contributes to the progression of obesity-related complications such as hyperglycemia, insulin resistance, and hepatic steatosis.^311^ Therefore, the regulation of mitophagy must be finely balanced to ensure optimal mitochondrial function and metabolic health.

#### Type 2 Diabetes

Type 2 diabetes mellitus (T2DM) is a prevalent metabolic disorder characterized by chronic hyperglycemia, insulin resistance, and impaired pancreatic β-cell function.^312^ The pathogenesis of T2DM is complex, involving multiple factors such as age, obesity, physical inactivity, oxidative stress, mitochondrial dysfunction, and chronic inflammation. Among these, mitochondrial dysfunction and mitophagy have emerged as critical players.^313^ PINK1–Parkin-mediated mitophagy is activated in the submandibular gland cells of diabetic mice.^314^ In peripheral blood mononuclear cells of individuals with T2DM, deregulated mitophagy is associated with the accumulation of dysfunctional mitochondria and increased ROS generation.^315^ Mutations in mitophagy-related genes, including *PRKN*, *PINK1*, *PDX1*, and *CLEC16A*, have been shown to contribute to the development of T2DM in both humans and mice.^316–318^ Growing evidence supports the necessity of fine-tuning mitophagic activity in β-cells to ensure their proper function.^308,319^

#### Metabolic Dysfunction-Associated Steatotic Liver Disease

MASLD is characterized by fat accumulation in the liver and is a major cause of chronic liver disease.^320^ Emerging evidence suggests that mitophagy plays a critical role in modulating hepatic lipid metabolism and mitigating MASLD progression.^321^ In the early stages of MASLD, compensatory activation of mitophagy serves as a protective mechanism to counteract lipid overload.^322,323^ Indeed, PINK1–Parkin-mediated mitophagy facilitates the removal of dysfunctional mitochondria to maintain energy balance and reduce oxidative stress, thus alleviating MASLD progression.^322^ This process is essential for the maintenance of mitochondrial integrity, as impairment of mitophagy by PINK1 or Parkin disruption contributes to increased oxidative stress, exacerbating hepatic steatosis and inflammation.^324^ In addition to the PINK1–Parkin pathway, alternative mitophagy receptors and regulators also contribute to mitochondrial quality control in MASLD. For example, sirtuin 3 (SIRT3), a nicotinamide adenine dinucleotide (NAD^+^)-dependent deacetylase, enhances BNIP3-dependent mitophagy via the ERK–CREB signaling axis, thereby attenuating apoptosis and lipid deposition in hepatocytes.^325^ Likewise, the KEAP1–Rbx1 complex, recruited to mitochondria by p62, promotes ubiquitination of damaged organelles, restoring mitophagic flux and mitigating MASLD in a Parkin-independent manner.^326^ These findings underscore the diversity of molecular mechanisms that govern mitophagy in hepatocytes.

### Cardiovascular Diseases

Cardiovascular diseases remain the leading cause of mortality worldwide, with mitochondrial dysfunction playing a pivotal role in their pathogenesis. As the primary energy producers of the heart, mitochondria are essential for maintaining cardiomyocyte function through ATP production, calcium homeostasis, and redox balance. Given the heart’s high metabolic demand, mitochondrial quality control mechanisms, particularly mitophagy, are crucial for cellular homeostasis.^327^ Dysregulation of mitophagy has been implicated in the pathogenesis of diverse cardiovascular disorders,^328^ including ischemia–reperfusion (I/R) injury, cardiomyopathy, myocardial hypertrophy, atherosclerosis, and heart failure.

#### Ischemia–Reperfusion Injury

I/R injury occurs when blood flow is restored to tissue following a period of ischemia, triggering mitochondrial calcium overload, mitochondrial permeability transition pore (mPTP) opening, and excessive ROS production.^329^ Mitophagy plays a key role in minimizing damage by clearing dysfunctional mitochondria, although its regulation may have varying effects at different stages of I/R injury. During ischemia, AMPK activation induces protective mitophagy to clear dysfunctional mitochondria.^330^ Both PINK1 and Parkin are upregulated during I/R injury, and this is necessary for the induction of ischemic preconditioning.^331^ By contrast, reperfusion disrupts this balance, resulting in increased mitochondrial fission and apoptosis. *Prkn*-knockout mice show disrupted mitophagy after I/R injury, leading to a larger infarct area, more severe heart damage, and decreased cell survival, emphasizing the role of Parkin in mitophagic clearance.^332^ Conversely, the inhibition of autophagy has been shown to reduce heart injury in certain cases. For example, *Beclin 1* heterozygous mice are protected against reperfusion damage by attenuating excessive autophagosome formation.^333^ These findings demonstrate that mitophagy acts as a double-edged sword, and requires precise modulation to avoid detrimental outcomes.

#### Heart Failure

Heart failure is a condition in which the heart is unable to pump blood effectively to meet the body’s needs. It can result from conditions such as hypertension, coronary artery disease, or cardiomyopathy. Mitochondrial dysfunction and cardiomyocyte apoptosis, often driven by insufficient mitophagy, are key contributors to heart failure progression.^329^ Biopsies of heart failure patients have revealed a modest decrease in the expression of autophagy-related markers such as LC3 and Beclin 1,^334^ together with a significant reduction in PINK1 levels.^335^ PINK1 knockout mice display impaired mitochondrial function, increased oxidative stress, cardiomyocyte apoptosis, and ventricular dysfunction.^335^ Moreover, as a key regulator of mitophagy, AMPKα exhibits isoform-specific effects on heart failure. AMPKα2 promotes mitophagy and prevents disease progression by phosphorylating PINK1, whereas a transition from the AMPKα2 isoform to the AMPKα1 isoform impairs mitophagy and accelerates heart failure.^336^ Consistent with these findings, MFN2 deficiency impairs mitochondrial fusion and Parkin recruitment, leading to cardiac hypertrophy and heart failure.^337,338^

## Therapeutic Interventions Targeting Mitophagy Pathways

Given the broad effects of dysregulated mitophagy in aging and age-related disorders, the modulation of mitophagy pathways has emerged as a promising therapeutic strategy. To date, most efforts have focused on enhancing mitophagy, as its deficiency contributes to multiple diseases and the restoration of mitophagy promotes mitochondrial health. However, the inhibition of mitophagy is also of interest in certain contexts, such as antiviral defense and cancer therapy.

### Mitophagy Activators

Mitophagy can be triggered by a variety of stimuli. Foremost among these bioactive molecules, the naturally occurring postbiotic compound UA is a prominent mitophagy inducer^339,340^ whose effects have been confirmed in humans.^341,342^ Thymol, a terpenoid abundant in essential oils derived from thyme, has recently been suggested to induce mitophagy through the PINK1–Parkin pathway.^343^ Nutritional interventions that replenish NAD^+^ through its precursors — nicotinamide riboside (NR), nicotinamide (NAM), and nicotinamide mononucleotide (NMN) — can enhance mitochondrial biogenesis and restore mitophagy.^344–349^ Similarly, the natural polyamine spermidine promotes both autophagy and mitophagy.^350–352^ Physical activity, including different types of exercise, has also been shown to boost mitophagy in both mice and humans, potentially via AMPK-mediated ULK1 phosphorylation.^353–355^ In addition, pharmacological agents such as the PARP inhibitor AZD2281 (also known as Olaparib)^356,357^ and the mTOR inhibitor rapamycin^358^ have been reported to induce mitophagy.

Most known modulators act on global autophagy, and only a few show preference for mitophagy, underscoring the need for truly selective modulators. The following section focuses on selective nutraceutical and pharmacological mitophagy activators, summarizing recent preclinical and clinical advances in the development of mitophagy-targeting interventions.

#### Urolithin A: A Natural Compound that Boosts MMtophagy and Mitochondrial Function

UA is a natural postbiotic compound produced by gut microflora through the metabolism of ingested polyphenols such as ellagitannins (ETs) and ellagic acid (EA), which are abundant in pomegranate, berries, and nuts.^359^ Approximately 40% of individuals can naturally convert dietary precursors into UA at variable levels.^360,361^ The benefits of UA supplementation were first described in *C. elegans* and mice^339^ and have since been confirmed in humans^210,340–342^ (Table 1). UA is now recognized as a mitochondrial modulator that enhances mitophagy, although its molecular target remains to be determined.^190^ Its mechanism involves the stabilization of PINK1, the accumulation of pUb,^210,339,340^ and potentially the activation of BNIP3.^210,340^ UA supplementation has demonstrated wide-ranging benefits across various models of aging and diseases. In *C. elegans*, it extends lifespan, and in mice, it improves health outcomes in neurodegenerative disorders, muscle dysfunction, cardiovascular diseases, and aging, with anti-inflammatory effects commonly observed across several preclinical human disease models. Human clinical trials have demonstrated that UA induces mitophagy in muscle, improves muscular function, ameliorates inflammation, and elevates plasma biomarkers of mitochondrial health.^342,360,362^ To date, UA remains the only clinically validated bioactive compound shown to enhance mitophagy in humans, highlighting its therapeutic potential as a proof of concept for mitophagy-targeted interventions.

**Table 1:** Clinical trials of Urolithin A.

Trial ID	Eligibility	Study Focus	Trial status/Key Findings
NCT02655393	Adults ≥ 61 and ≤ 85 years of age, BMI ≥ 18 and ≤ 32 kg/m^2^.	Safety, pharmacokinetic and pharmacodynamic assessment	Completed. UA is safe and bioavailable in humans. UA modulated muscle and mitochondrial biomarkers.^341^
NCT04160312	Adults ≥ 18 and ≤ 80 years of age.	Bioavailability	Completed. Direct supplementation with UA significantly increased plasma levels of UA.^361^
NCT06362018/NCT06853197	Adults ≥ 8 and ≤ 45 years of age, BMI ≥ 18.5 and ≤ 30 kg/m^2^.	Bioavailability	Completed. Results pending*.
NCT03464500	Adults ≥ 40 and ≤ 65 years of age, BMI ≥ 25 and ≤ 34.9 kg/m^2^.	Muscle function	Completed. Improved muscle strength, peak O_2_ consumption, 6 min walk distance, reduced plasma inflammation marker.^342^
NCT03283462	Adults ≥ 65 and ≤ 90 years of age.	Muscle function	Completed. Improved muscle endurance measured by the number of muscle contractions until fatigue, reduced plasma inflammation marker.^379^
NCT04783207	Male adults ≥ 18 and ≤ 40 years of age, elite and sub-elite endurance runners.	Muscle function	Completed. Reduced rates of perceived exertion and indirect markers of post-exercise muscle damage, improved maximal O_2_ consumption. Did not further enhance performance in highly trained endurance runners.^380^
NCT06556706	Frail adults ≥ 65 and ≤ 85 years of age, BMI ≥ 18 and ≤ 35 kg/m^2^.	Mitochondrial quality in muscle	Completed. Results pending*.
NCT05735886	Adults ≥ 45 and ≤ 70 years of age, BMI ≤ 34.9 kg/m^2^.	Mitochondrial activity in immune cells, immune function	Completed. UA elicits immune remodeling, characterized by changes in mitochondrial measurements and immune markers.^377^
NCT05921266	Obese adults ≥ 40 and ≤ 64 years of age, BMI ≥ 30 kg/m^2^.	Endothelial and cerebrovascular function	Completed.^382^ Results pending*.
NCT06022822	Males ≥ 18, with confirmed prostate cancer undergoing radical prostatectomy.	Oxidative stress in tumor tissue	Recruiting*.
NCT06274749	Adults ≥ 55 and ≤ 64 years of age, BMI ≥ 27 kg/m^2.^	Insulin levels and glucose tolerance	Recruiting*.
NCT06324214	Adults > 40 years of age, with ≥ 10 pack-years of smoking history, with chronic obstructive pulmonary disease (COPD), during pulmonary rehabilitation.	Exercise endurance capacity	Recruiting*.
NCT07161310	Adults ≥ 18 years of age, with untreated solid cancer and planned Immune checkpoint inhibitor therapy.	Immune system	Not yet recruiting*.
NCT06990256	Adults ≥ 45 and ≤ 70 years of age, with Pittsburgh Sleep Quality Index > 5.	Sleep quality, aging markers	Not yet recruiting*.
NCT07060898	Adults ≥ 40 years of age.	Cognitive function	Active*.

*Status as of November 30, 2025; only ClinicalTrials.gov entries.

##### Neurodegenerative Disorders

UA targets mitochondrial dysfunction and inflammation — key drivers of neurodegenerative disorders^363 ^— positioning mitophagy enhancement as a novel therapeutic approach for PD and AD. In 6-hydroxydopamine (6-OHDA)-induced and neurotoxin MPTP (1-methyl-4-phenyl-1,2,3,6-tetrahydropyridine)-induced mouse models of PD, UA improved motor dysfunction, alleviated dopaminergic neurotoxicity,^364,365^ and reduced neuroinflammation.^364^ In an APP/PS1 mouse model of AD that carries human AD-linked mutations in *APP* and *PSEN*1 genes, UA restored neuronal mitophagy, ameliorated cognitive decline, reduced Aβ pathology, and mitigated neuroinflammation.^210,366^ Similar benefits were observed in models of AD with tau pathology or DNA repair-deficiency, in which UA reduced tau pathology and DNA damage.^367,368^ The neuroprotective effects of UA extend to other CNS disorders. UA alleviated locomotor symptoms in a zebrafish model of ALS that expresses glycine-proline dipeptide repeats in a *c9orf72* knockout context.^226^ In a mouse model of ALS carrying the human *SOD1* G93A mutation and exposed to copper, UA activated mitophagy, reduced neuroinflammation, and improved muscle atrophy and motor dysfunction.^369^ In the experimental autoimmune encephalomyelitis (EAE) model of multiple sclerosis, UA reduced demyelination and inflammation.^370^ The potential effects of UA on cognitive function in humans are being investigated (NCT07060898).

##### Immune Function

UA directly modulates the function and status of multiple immune cell types, including macrophages,^371–373^ dendritic cells,^370^ and T cells.^287,370,374–377^

In addition to the brain,^210,366,368,370^ UA exerts anti-inflammatory actions in muscle,^339,340^ liver, and adipose tissue,^378^ as well as systemically.^342,377^ In macrophages, UA suppresses LPS-induced NF-κB activation, ROS generation, and proinflammatory cytokines such as IL-1β, IL-6, and tumor necrosis factor-α (TNF-α),^371–373^ thereby inhibiting M1 polarization. Similarly, UA restrains the activation of dendritic cells and microglia and prevents the differentiation of CD4^+^ T cells into T helper 17 cells (Th17) and the infiltration of T cells into the CNS, suppressing the progression of experimental autoimmune encephalomyelitis (EAE) in vivo.^370^

On the other hand, UA increases the number of CD8^+^ T_SCM_, a subset of minimally differentiated T cells. In the tumor microenvironment, such expansion of T_SCM_ confers superior CD8^+^-mediated anti-tumor immunity and facilitates the production of potent chimeric antigen receptor (CAR) T_SCM_.^287^ Consistent with these observations, UA enhances the persistence and effector functions of CD8^+^ cytotoxic T lymphocytes (CTLs) and human CAR T cells^376^ and promotes CD8^+^ T cell-dependent cancer immunosurveillance. In aged mice, UA boosts hematopoietic capacity, leading to the restoration of effector CD8^+^ T cell differentiation and function, thus improving viral control.^374^

The context-dependent immunomodulatory effects of UA await further exploration. Its effects on the human immune system have been investigated in a clinical trial (NCT05735886). It was suggested that short-term UA supplementation modulates human immune cell composition and function. For example, UA expanded peripheral naive-like, less terminally exhausted CD8^+^ cells, while increased CD8^+^ fatty acid oxidation capacity.^377^ Its effects on cancer therapy are also being investigated (NCT06022822, NCT07161310). These findings highlight UA as an immunometabolic modulator with potential in both autoimmune and cancer immunotherapy contexts.

##### Muscle Function

UA consistently improves muscle health. In middle-aged mice on an HFD, 8 months of UA supplementation improved spontaneous exercise and grip strength, and 6 weeks of treatment boosted running endurance in aged mice.^339^ Young rats also showed increased running capacity after 2 weeks of UA.^339^ In Duchenne muscular dystrophy (DMD) models, UA rescued mitophagy and mitochondrial respiratory capacity in muscle fibers and muscle stem cells, reduced inflammation, and improved muscle integrity and function.^340^ UA also reduced inflammation in the diaphragm and increased the survival rate.^340^

Double-blind, randomized, placebo-controlled human clinical trials reinforce these findings.^362^ In middle-aged individuals with high BMI (NCT03464500), 4 months of UA supplementation significantly improved muscle strength and led to clinically relevant improvements in peak oxygen consumption and 6-min walk distance, together with reduced plasma inflammatory markers and increased phosphorylated Parkin in skeletal muscle.^342^ In healthy elderly individuals (NCT03283462), UA significantly improved muscle endurance.^379^ A trial studying the effect of UA on muscle performance and endurance in elite and sub-elite endurance runners (NCT04783207) showed that UA supplementation significantly lowered ratings of perceived exertion and reduced indirect markers of post-exercise muscle damage, although running performance was not significantly improved.^380^ Another ongoing study is investigating the effects of UA on mitochondrial quality in the muscle of frail elderly individuals (NCT06556706), which will provide further insight into its therapeutic potential for muscle-related dysfunctions.

##### Cardiovascular and Metabolic Function

UA demonstrates protective effects against cardiovascular dysfunction. In DMD models, it reduced cardiac muscle fibrosis and hypertrophy.^340^ In HFD-fed mice, UA prevented diastolic dysfunction and cardiac remodeling.^381^ In a rat myocardial infarction model, post-surgery UA administration restored mitochondrial gene expression and improved systolic function.^328^ In addition, UA improved diastolic cardiac function in aged mice^328^ and reduced plasma ceramides linked to cardiovascular disease risk in both mice and humans.^328^ A recently completed clinical trial (NCT05921266) will reveal the effect of UA on cerebral blood flow in middle-aged adults with obesity.^382^

In HFD-fed mice, UA prevented obesity, liver steatosis, systemic inflammation, glucose intolerance,^383^ and insulin resistance.^378,383^ In therapeutic settings, UA also reversed obesity and restored glucose homeostasis.^383^ Similar anti-obesity effects were observed in the *ob/ob* genetic mouse models of obesity.^383^ An ongoing clinical trial (NCT06274749) is investigating whether UA supplementation can improve insulin levels and glucose tolerance in overweight elderly adults.

#### Spermidine: A Natural Polyamine With Pleiotropic Effects

The naturally occurring polyamine spermidine is found ubiquitously in various organisms, including humans. It is abundant in foods such as wheat germ, soybeans, nuts, and certain fruits and vegetables. In addition to dietary sources, spermidine is also synthesized through intracellular biosynthesis and by the gut microbiota.^352^ It plays crucial roles in cellular processes, including DNA and RNA stabilization, oxidative stress regulation, cell proliferation, apoptosis, and immune regulation.^384,385^

Tissue spermidine levels decline with aging,^386,387^ and spermidine supplementation has been shown to improve cellular health.^352^ Notably, spermidine administration extends both healthspan and lifespan in flies, worms, and mice by inducing autophagy.^350,386^ Its bioavailability^388^ (NCT06017219, NCT05017428), biological effects (NCT05459961, NCT04823806), and potential association with mortality^389^ (NCT03378843) are active areas of clinical research (Table 2).

**Table 2:** Clinical trials of Spermidine.

Trial ID	Eligibility	Study Focus	Trial status / Key Findings
NCT06017219	Male adults ≥ 18 and ≤ 70 years of age, BMI ≥ 18.5 and ≤ 28 kg/m^2^.	Bioavailability	Active*.
NCT05017428	Adults ≥ 20 and ≤ 40 years of age, BMI ≥ 19 and ≤ 27 kg/m^2^.	Bioavailability	Completed. Spermidine intake prevented the decrease in its concentrations in the control arm group.^392^
NCT03378843	Adults ≥ 40 and ≤ 79 years of age in 1990.	Association with mortality	Completed. Nutrition rich in spermidine is linked to increased survival in humans.^388^
NCT04823806	Adults ≥ 18 and ≤ 75 years of age, BMI ≥ 17 and ≤ 40 kg/m^2^.	Multi-level molecular response	Unknown*.
NCT05459961	Caucasian male adults ≥ 50 and ≤ 70 years of age.	Metabolic response	Active*.
NCT05128331	Adults ≥ 50 and ≤ 100 years of age, with diastolic heart failure.	Metabolic, neurological-cognitive, and cardiovascular function	Unknown*.
NCT06186102	Adults ≥ 65 years of age, with coronary artery disease and carries risk factors.	Cardiovascular, muscular, metabolic, and physical function, and inflammation	Recruiting*.
NCT04405388	Adults ≥ 19 and ≤ 99 years of age, with persistent arterial hypertension.	Blood pressure	Unknown*.
NCT03094546	Adults ≥ 60 and ≤ 90 years of age.	Cognitive function	Completed. Higher dietary spermidine intake was positively associated with several structural brain measures.^393,394^
NCT04138134	Adults ≥ 18 and ≤ 85 years of age, with venous dysfunction.	Venous function upon autophagy activation	Unknown*.
NCT05421546	Adults ≥ 60 and ≤ 90 years of age.	Vaccination upon autophagy activation	Completed. Results pending*.

*Status as of November 30, 2025; only ClinicalTrials.gov entries.

Spermidine also stimulates mitophagy^350^ by triggering mitochondrial depolarization, enhancing PINK1 accumulation and Parkin translocation to damaged mitochondria^351^; the ataxia telangiectasia mutated protein (ATM) drives the initiation of this mitophagic cascade.^351^ Spermidine-induced mitophagy has been observed in various tissues, with significant physiological benefits. In geriatric mice, spermidine administration restores mitophagy in muscle stem cells (satellite cells), reversing senescence and rejuvenating regenerative functions.^390^ In D-gal-induced aging rats, spermidine reduces damaged, swollen, and fused mitochondria in skeletal muscle, suggesting enhanced clearance of dysfunctional mitochondria via mitophagy.^391^ In addition, spermidine promotes follicle development, oocyte maturation, early embryonic development, and fertility in aged female mice.^392^ In cardiomyocytes, it induces mitophagy in both young and aged cells, improving cardiomyocyte health.^350^

These findings underscore the potential of spermidine as a nutraceutical, prompting ongoing clinical trials. One study is evaluating spermidine treatment in elderly patients with coronary artery disease (NCT06186102), and another is investigating its role in enhancing exercise training outcomes in patients with heart failure (NCT05128331). Orally supplemented spermidine crosses the blood–brain barrier (BBB), promotes cerebral mitochondrial quality control, and enhances hippocampal mitochondrial function, leading to improved spatial and temporal memory in aged mice.^393^ In humans, higher dietary spermidine intake is correlated with enhanced cognitive function.^393^ A clinical trial investigating a polyamine-rich diet in individuals with subjective cognitive decline (NCT03094546) found a positive association between higher spermidine intake and several brain structural measures, including cortical thickness and hippocampal volume.^394,395^

Although spermidine exhibits a wide range of beneficial effects in addition to mitophagy,^352^ the extent to which mitophagy activation underlies these broader benefits remains to be clarified. Further research is needed to determine whether mitophagy is a primary driver of these effects.

#### Activation of the PINK–Parkin Mitophagy Pathway with New Chemical Entities

**Fig. 4:**
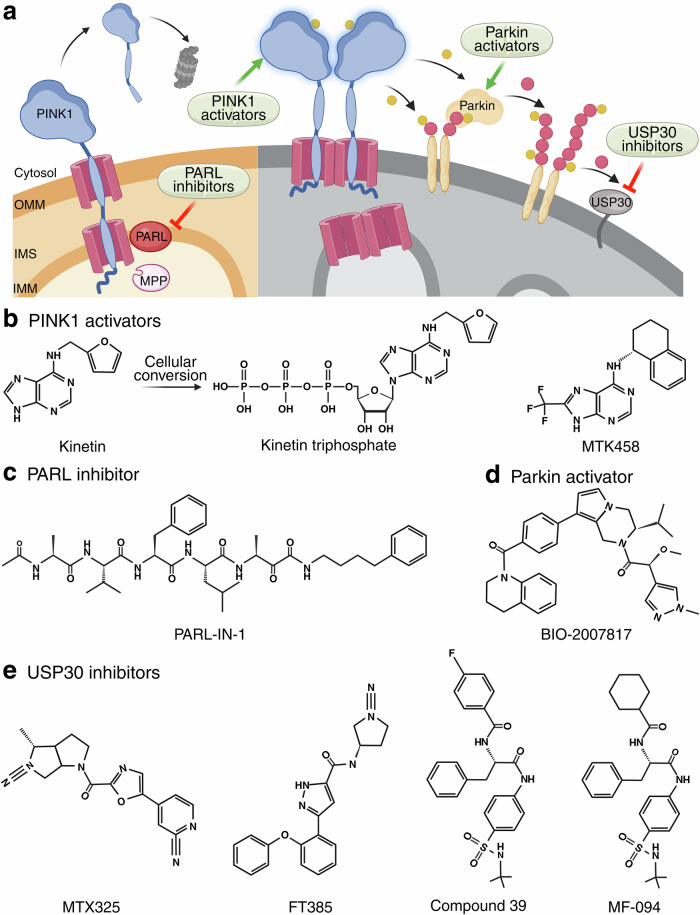
Selective mitophagy inducers targeting the PINK1–Parkin pathway. **a** Schematic overview of mitophagy activators and their targets. **b** Structure of PINK1 activators. **c** Structure of a PARL inhibitor. **d** Structure of a Parkin activator. **e** Structure of USP30 inhibitors.

The enhancement of mitophagy shows broad therapeutic potential, as demonstrated by the beneficial effects of UA and spermidine. Advances in mitophagy research have led to the development of targeted therapies, often using new chemical entities (NCEs), particularly for PD.^190,396^ Here, we summarize emerging pharmaceutical strategies aimed at boosting PINK1–Parkin-mediated mitophagy (Fig. 4).

##### PINK1 Activators

PINK1 serves as the gatekeeper of the PINK1–Parkin mitophagy pathway, and its stabilization and accumulation on depolarized mitochondria are a crucial step in the initiation of mitophagy.^38–40^ One approach to PINK1 activation involves the design of neo-substrates that directly increase its activity. The ATP analog kinetin triphosphate (KTP) was shown to increase the catalytic activity of both wild-type and G309D mutant PINK1^397^ independently of mitochondrial depolarization.^398^ However, long-term administration of its precursor, kinetin, which can be taken up by cells and converted to KTP, failed to prevent αSyn-induced neurodegeneration in PD rodent models. This was likely due to its low potency, poor pharmacokinetics, and limited brain penetrance.^399^ Moreover, recently reported cryogenic electron microscopy structures revealed that KTP does not bind human wild-type PINK1.^400^

Follow-up work has led to the design, synthesis, and characterization of additional kinetin derivatives.^401–403^ In the exploration of N^6^-substituted adenines and adenosines, many of the nucleoside analogues were able to activate PINK1, as determined by the phosphorylation of Parkin.^401,402^ The effort to discover novel small-molecule PINK1 activators with drug-like properties led to the development of the kinetin derivative MTK458 (WO2021168446A1)^403^ by Mitokinin (now part of AbbVie). MTK458 induces mitophagy in the presence of the mitochondrial stressors, FCCP and oligomycin, while exhibiting low mitochondrial toxicity, favorable oral pharmacokinetics, and high brain penetrance.^403^ MTK458 binds to PINK1 and stabilizes the PINK1–TOM complex, promoting the clearance of accumulated pUb and αSyn pathology both in vitro and in vivo.^403^ MTK458, now named ABBV-1088, is currently in a Phase I clinical trial to evaluate its single-dose safety, tolerability, and pharmacokinetic properties in healthy adults (NCT06414798). Further studies are planned to assess multiple ascending doses, drug–drug interactions, and pharmacokinetics in Asian populations (NCT06579300).

Thymol, a natural compound, has also been reported to activate PINK1-mediated mitophagy in *C. elegans*, zebrafish, and mice.^343^ Thymol lowers mitochondrial membrane potential, inhibits mitochondrial respiration, and increases mtROS, with effects that are transient and reversible. Functionally, thymol prevents liver fat accumulation in HFD-fed mice, and in *C. elegans*, it enhances thermotolerance in a PINK1-dependent manner.^343^ Further characterization of thymol-mediated mitophagy may inform novel strategies for PINK1 activation.

##### PARL Inhibitors

In healthy mitochondria, mitochondria-localized PINK1 is processed by MPP and PARL before being degraded by the proteasome.^31–37^ Targeting PARL represents an alternative approach for stabilizing PINK1. A pilot study identified N-substituted peptidyl α-ketoamides as selective inhibitors of rhomboid proteases, acting at nanomolar concentrations by covalently and reversibly binding to the catalytic serine at its active site, mimicking its substrates.^404^ Building on this, a ketoamide inhibitor of PARL, PARL-IN-1, was developed by modifying a fragment of the human PINK1 sequence located at the PARL cleavage site and incorporating a phenyl-butyl substituent at its amidic nitrogen.^405^ PARL-IN-1 effectively inhibits PARL activity in vitro, with a half-maximal inhibitory concentration (IC_50_) of 28 nM. In cells, PARL-IN-1 has a considerable effect on PARL inhibition, as endogenous PINK1 remains largely uncleaved and is stabilized in its 66 kDa form.^405^ Stabilized PINK1 further recruits Parkin to the mitochondria without affecting mitochondrial membrane potential. However, PARL inhibition by PARL-IN-1 reveals alternative PINK1 trafficking pathways, leading to the accumulation of MPP-cleaved PINK1 in the mitochondria and partial degradation of PINK1 by the IMM metalloprotease OMA1.^405^

##### Parkin Activators

Parkin is the major amplifier of PINK1–Parkin-mediated mitophagy.^60,61^ To screen for Parkin activators, Biogen developed a high-throughput screening system using a Parkin autoubiquitination in vitro reaction system combined with a time-resolved Förster resonance energy transfer (TR-FRET) assay. Structure–activity relationship (SAR) studies were performed to optimize the original hits, and this led to a potent lead candidate, BIO-2007817.^406^ Interestingly, BIO-2007817 is not a direct activator of Parkin, since it has a low affinity for Parkin in the absence of pUb,^407^ and it does not increase Parkin’s translocation to mitochondria or affect mitophagy in cells.^406^ Instead, BIO-2007817 amplifies the existing activation mechanism, acting as a positive allosteric modulator (PAM) to activate Parkin in a regulated manner.^407,408^ BIO-2007817 functions as a molecular glue, enhancing the ability of pUb to activate Parkin by releasing the catalytic RING2 domain from RING0, shifting Parkin toward its active conformation in a manner analogous to Parkin activation by phosphorylation.^407^ BIO-1975900 is closely related to BIO-2007817, and the two compounds bind to Parkin at the same location. Crystallography revealed the binding of BIO-1975900 to the RING0 domain of Parkin, close to pUb, making contacts with both Parkin and pUb.^407^ This mechanism enables BIO-2007817 to specifically rescue ubiquitination and mitophagy of pathological *PRKN* variants that lack a functional Ubl domain, such as R42P and V56E.^407^

##### USP30 Inhibitors

DUBs are endogenous proteins that antagonize Parkin-mediated mitophagy,^62–64,409^ and inhibition of DUBs could promote mitophagy,^410^ making them attractive therapeutic targets.^411^ Several DUBs, including USP30,^62^ USP15,^412^ and USP8,^413^ have been shown to negatively regulate mitophagy. USP30 is distinguished from other DUBs by its localization on the OMM^62,414^ and has been a focus of intensive inhibitor development.

Mission Therapeutics has described covalent inhibitors of USP30, including N-cyanopyrrolidine-based MTX325 (also known as MTX115325), which exhibits good oral bioavailability and CNS penetration (WO2021249909A1).^415^ Treatment with MTX325 for 24 hours or 7 days upregulated TOM20 ubiquitylation in iPSC-derived dopaminergic neurons.^415^ In an AAV1/2-A53T-SNCA αSyn overexpression PD mouse model that shows dopaminergic neurodegeneration and motor deficits, 10 weeks of oral MTX325 treatment protected mice against αSyn-induced loss of TH-positive neurons and abrogated the depletion of dopamine and its metabolites.^415^ MTX325 is currently under a Phase I clinical trial in healthy volunteers and patients (ISRCTN20898392). Another Mission Therapeutics USP30 inhibitor, MTX652 (also known as MTX115652), promotes the ubiquitination of TOM20 in cells and protects mice from transverse aortic constriction-induced cardiac hypertrophy and left ventricular dysfunction.^416^ MTX652 has finished a Phase I clinical trial (EudraCT number: 2021-006764-24)^417^ and has received FDA clearance for a Phase II clinical trial. FT385 is an N-cyanopyrrolidine-modified USP30 inhibitor (WO2019071073A1) from FORMA Therapeutics (now part of Novo Nordisk). FT385-mediated USP30 inhibition phenocopies *USP30* knockout in enhancing basal mitophagy and promoting TOM20 ubiquitination upon mitochondrial depolarization.^418^

Compounds from other structural classes have also been shown to inhibit USP30. For example, the diterpenoid derivative 15-oxospiramilactone (S3) activates mitochondrial fusion and restores the mitochondrial network via USP30 inhibition.^419^ Starting from a racemic phenylalanine derivative, Mitobridge (now part of Astellas) performed SAR studies and developed several potent, non-covalent, specific inhibitors of USP30, such as MF-094.^420^ Another Mitobridge entity, compound 39 from their benzenesulfonamide series, enhances TOM20 ubiquitination following mitochondrial depolarization and restores mitophagy in patient-derived iPSC dopaminergic neurons.^421^ In addition, a novel peptide derived from the transmembrane domain of USP30 can directly target USP30 through an allosteric autoinhibition mechanism, thereby increasing mitophagy.^422^ Further preclinical investigations are needed to verify their therapeutic potential in the treatment of neurodegenerative disorders.

In addition, USP30 depletion enhances CCCP- and BH3 mimetic-induced cell death.^64^ This indicates that USP30 inhibition, in addition to promoting mitophagy in neurodegeneration, may potentially serve as a target for cancer therapy. Consistent with this notion, a recent study suggests that USP30 is upregulated in the liver of mice fed with HFD and treated with N-nitrosodiethylamine (DEN), as well as in human HCC samples. Overexpressing a stabilized form of USP30 promoted tumor growth in DEN/CCl_4_-induced HCC, whereas USP30 knockout reduced tumorigenesis in mice.^423^ Furthermore, it has been shown that mitophagy is suppressed during T cell exhaustion; knockout of USP30 or inhibition of USP30 by the specific inhibitor ST-539^424^ rejuvenates effector function in exhausted CD8^+^ T cells, enhancing their antitumor immunity.^305^ Together, these findings provide proof of concept that USP30 inhibition may be exploited not only for neurodegenerative disorders but also for cancer therapy.

#### Activation of Alternative Mitophagy Pathways

In addition to targeting the PINK1–Parkin pathway, targeting alternative mitophagy pathways has also yielded promising preclinical outcomes. For instance, induced myeloid leukemia cell differentiation protein 1 (MCL1) is an anti-apoptotic BCL-2 family protein. The MCL1 isoform, located in the mitochondrial matrix, is an important mitochondrial homeostasis regulator^425^ and a putative mitophagy receptor,^426,427^ although conflicting results have been reported regarding its function.^428^ The FDA-approved BH3 mimetic UMI-77, which targets MCL1, has been shown to activate mitophagy without causing mitochondrial damage or apoptosis.^429^ Notably, this effect occurs independently of Parkin or selective autophagy receptors.^429^ Most importantly, intraperitoneal injection of UMI-77 induces mitophagy in the mouse brain, improving the learning and memory of APP/PS1 mice.^429^

Another novel mitophagy inducer is mitophagy-inducing coumarin (MIC),^430^ a benzocoumarin compound that is abundant in various edible vegetables and plants. MIC enhances TFEB (HLH-30 in *C. elegans*) expression and lysosomal function by inhibiting ligand-induced activation of the nuclear hormone receptor DAF-12 (in *C. elegans*)/FXR (in mammals), thereby triggering mitophagy. MIC robustly extends lifespan in *C. elegans* and alleviates mitochondrial dysfunction in mammalian cells, highlighting its therapeutic potential in aging and age-related diseases. Interestingly, emerging evidence suggests that UA, like MIC, may also modulate FXR activity.^430^ Determining whether DAF-12/FXR directly regulates UA-mediated mitophagy could provide deeper mechanistic insight into the efficacy of UA.

New small-molecule mitophagy boosters are also entering development. Capacity Bio has developed CAP-1902, a small-molecule mitophagy booster that functions as an agonist of the Mas-related G-protein coupled receptor (WO2022165189A1), and its effects on the brain, cardiac tissue, liver, and muscles are being investigated.^431,432^ Furthermore, Vandria has developed VNA-318, another small-molecule mitophagy inducer designed for mild cognitive impairment (WO2024025953A2), which is undergoing a Phase I clinical trial in healthy males (NCT06721091). These advances underscore the therapeutic promise of activating mitophagy through alternative druggable pathways in addition to the PINK1–Parkin cascade.

### Mitophagy Inhibitors

Compared with the extensive research on mitophagy activators, efforts to identify mitophagy inhibitors remain preliminary. Nevertheless, several candidate molecules and pathways have been described.

The fission inhibitor Mdivi-1 is widely used as a mitophagy inhibitor.^270^ In dopaminergic neuronal cell lines, Mdivi-1 counteracts the overall fission effect caused by mutant human *PINK1*.^433^ It has also been shown to inhibit BCL2L13-mediated mitophagy, thereby reducing the migration and invasion of glioblastoma cells.^141^

Another inhibitory regulator is PTEN-long (PTEN-L), an N-terminal extended isoform of PTEN that localizes to the OMM.^434^ PTEN-L acts as a protein phosphatase for ubiquitin, dephosphorylating pUb at Ser65. This prevents Parkin recruitment and subsequent phosphorylation, thereby inhibiting FCCP- or Oligomycin-induced mitophagy.^435^ In SH-SY5Y cells, prion protein peptide treatment induces PTEN-L accumulation and mitochondrial localization, which in turn exacerbate mitophagy dysfunction and promote cell apoptosis. Further investigation of PTEN-L’s mechanism of action may clarify its role in disease modulation and guide drug development.

The small molecule IGS2.7 has also been identified as a mitophagy inhibitor. In U2OS cells, IGS2.7 was shown to inhibit deferiprone-induced mitophagy, and in ARPE-19 cells with high levels of basal mitophagy, IGS2.7 further inhibits basal mitophagy. Mechanistically, IGS2.7 inhibits ULK1 activity. In cell and mouse models of ALS, IGS2.7 restores autophagy and mitochondrial protein levels to control levels.^436^ However, IGS2.7 also targets casein kinase 1, complicating its use as a basis for the development of selective mitophagy inhibitors.

## Concluding Remarks

Mitophagy plays an essential role in maintaining cellular homeostasis by selectively eliminating damaged or surplus mitochondrial components, and its dysregulation is increasingly recognized as a prominent contributor to various diseases. Over the past two decades, starting from the discovery of PD-associated PINK1 and Parkin, significant progress has been made in deciphering the molecular mechanisms that underlie mitophagy — from ubiquitin-mediated pathways to receptor- and lipid-mediated mechanisms, as well as emerging concepts such as piecemeal mitophagy. Mounting evidence indicates that mitophagy is highly context-dependent and engages distinct molecular pathways, but how these pathways coordinate mitochondrial turnover in different cell populations under physiological and pathological conditions remains unclear. Advances in mitophagy detection techniques, such as fluorescent reporters like *mito*-QC,^87^ mt-Keima,^89,437^ and mito-SRAI,^438^ will accelerate progress in this field. Integrating these tools with genetic, pharmacological, and single-cell and spatial omics approaches promises to further advance our understanding of mitophagy, thereby directing the development of specific and selective mitophagy-targeting therapies across diverse disease scenarios.

Recent translational efforts have identified promising mitophagy modulators, particularly activators, from natural compounds, such as UA, to novel chemical entities with improved pharmacological profiles. Optimizing their specificity, bioavailability, and safety for long-term use remains a major challenge. Importantly, mitophagy inhibition can also provide therapeutic benefits in settings such as infection-driven inflammation and cancer therapy, underscoring the need to consider these aspects when developing activators and to explore selective inhibitors. Moving forward, elucidating context-specific mitophagic alterations, their underlying pathways, and their in vivo relevance will be critical. Such insights should pave the way for cell type- and pathway-selective modulation of mitophagy, potentially offering more precise and safer therapeutic strategies across a wide spectrum of diseases.

## Supplementary information

Supplementary information, Table S1
